# Long-Term Effects of Empagliflozin in Patients with Chronic Kidney Disease

**DOI:** 10.1056/NEJMoa2409183

**Published:** 2024-10-25

**Authors:** William G. Herrington, Natalie Staplin, Nikita Agrawal, Christoph Wanner, Jennifer B. Green, Sibylle J. Hauske, Jonathan R. Emberson, David Preiss, Parminder Judge, Doreen Zhu, Rejive Dayanandan, Ryoki Arimoto, Kaitlin J. Mayne, Sarah Y.A. Ng, Emily Sammons, Michael Hill, Will Stevens, Karl Wallendszus, Susanne Brenner, Alfred K. Cheung, Zhi-Hong Liu, Jing Li, Lai Seong Hooi, Wen Liu, Takashi Kadowaki, Masaomi Nangaku, Adeera Levin, David Z.I Cherney, Aldo P. Maggioni, Roberto Pontremoli, Rajat Deo, Shinya Goto, Xavier Rossello, Katherine R. Tuttle, Dominik Steubl, Dan Massey, Martina Brueckmann, Martin J. Landray, Colin Baigent, Richard Haynes

**Affiliations:** Renal Studies Group, Clinical Trial Service Unit and Epidemiological Studies Unit, Nuffield Department of Population Health, University of Oxford, UK; Renal Studies Group, Clinical Trial Service Unit and Epidemiological Studies Unit, Nuffield Department of Population Health, University of Oxford, UK; University Clinic of Würzburg, Germany; Duke Clinical Research Institute, Durham, North Carolina, US; Boehringer Ingelheim International; Vth Department of Medicine, University Medical Center Mannheim, University of Heidelberg, Mannheim, Germany; Renal Studies Group, Clinical Trial Service Unit and Epidemiological Studies Unit, Nuffield Department of Population Health, University of Oxford, UK; Renal Studies Group, Clinical Trial Service Unit and Epidemiological Studies Unit, Nuffield Department of Population Health, University of Oxford, UK; Renal Studies Group, Clinical Trial Service Unit and Epidemiological Studies Unit, Nuffield Department of Population Health, University of Oxford, UK; University Clinic of Würzburg, Germany; University of Utah, Salt Lake City, US; National Clinical Research Center of Kidney Diseases, Jinling Hospital, Nanjing University School of Medicine, Nanjing, China; Fuwai Hospital, Chinese academy of Medical Sciences, National Center for Cardiovascular Diseases, Beijing, China; Hospital Sultanah Aminah, Johor Bahru, Malaysia; The University of Tokyo School of Medicine/ Toranomon Hospital; The University of Tokyo School of Medicine, Tokyo, Japan; University of British Columbia, Vancouver, Canada; University of Toronto, Canada; ANMCO Research Center, Florence, Italy; Università degli Studi and IRCCS Ospedale Policlinico San Martino di *Genova*, Italy; University of Pennsylvania Perelman School of Medicine, Philadelphia, US; Tokai University School of Medicine, Isehara, Japan; Hospital Universitario Son Espases, Health Research Institute of the Balearic Islands (IdISBa), Universitat Illes Balears (UIB), Palma de Mallorca, Islas Baleares, Spain; Providence Health Care and University of Washington, US; Boehringer Ingelheim International; Department of Nephrology, Hospital Rechts der Isar, Technical University of Munich, Germany; Elderbrook Solutions GmbH on behalf of Boehringer Ingelheim Pharma GmbH & Co.KG; Boehringer Ingelheim International; First Department of Medicine, Faculty of Medicine Mannheim, University of Heidelberg, Mannheim, Germany; Renal Studies Group, Clinical Trial Service Unit and Epidemiological Studies Unit, Nuffield Department of Population Health, University of Oxford, UK; Renal Studies Group, Clinical Trial Service Unit and Epidemiological Studies Unit, Nuffield Department of Population Health, University of Oxford, UK

## Abstract

**Background:**

Empagliflozin exerted positive cardiorenal effects in the EMPA-KIDNEY trial; this study reports active trial plus post-trial data.

**Methods:**

In this trial, patients with an estimated glomerular filtration rate 20-<45; or 45-<90mL/min/1.73m^2^ and urinary albumin-to-creatinine ratio of ≥200mg/g received either empagliflozin 10mg daily or matching placebo for two years (median). Subsequently, surviving participants who consented were observed post-trial for two additional years. No study drug was issued in the post-trial period, but local doctors could prescribe SGLT2 inhibitors. The primary composite outcome was kidney disease progression or cardiovascular death assessed from the start of the active trial to the end of the post-trial period.

**Results:**

4891 of 6609 randomized participants (74%) entered the post-trial period, during which, SGLT2 inhibitor use was similar between groups (empagliflozin group 43% vs. placebo 40%). Over the entirety of follow-up (study initiation to post-trial observation end) a primary outcome occurred in 865/3304 (26.2%) of the empagliflozin group and 1001/3305 (30.3%) of the placebo group (HR=0.79, 95%CI 0.72-0.87). There was a 13% (0.87, 0.76-0.99) reduction in risk of the primary outcome during the post-trial period in the empagliflozin group. Compared with placebo, original allocation to empagliflozin reduced risk of kidney disease progression (23.5%vs.27.1%), the composite of death or end-stage kidney disease (16.9%vs.19.6%), and cardiovascular death (3.8%vs.4.9%). There was no effect on non-cardiovascular death (5.3%vs.5.3%).

**Conclusions:**

In a broad range of patients with chronic kidney disease, empagliflozin continued to exert additional cardiorenal benefits for up to 12 months after it was discontinued. **(Funding:** Boehringer Ingelheim & others. **Trial registration numbers:** Clinicaltrials.gov: NCT03594110; EuDRACT: 2017-002971-24.)

Slowing chronic kidney disease (CKD) progression and avoiding end-stage kidney disease (ESKD, i.e., the need for dialysis or kidney transplantation) is highly desirable, given the associated adverse effects on quality of life, cardiovascular morbidity and mortality, and high economic costs.^1,2^ The EMPA-KIDNEY trial was established to assess the efficacy and safety of sodium glucose co-transporter-2 (SGLT2) inhibition with empagliflozin in a broad range of patients with CKD at risk of progression. Findings from the active part of this trial and other large trials provided compelling evidence that SGLT2 inhibitors substantially slowed kidney disease progression and reduced cardiovascular risk.^3–6^ SGLT2 inhibitors also reduced risk of hospitalization for heart failure and acute kidney injury in patients with CKD and other high-risk conditions (including diabetes and heart failure).^4^

Post-trial follow-up tests how benefits evolve once participants stop study drug, as it is possible that additional cardiorenal benefits or harms could emerge after its discontinuation. Our trial was relatively short, as it was stopped early for efficacy after a median of two years of follow-up. Consequently, there were a lower number of primary outcomes in participants who progressed more slowly, and a low number of ESKD and fatal outcomes.^3^ Post-trial follow-up provides particular value through prospectively collecting more ESKD outcomes, as these outcomes take longer to accrue than surrogates of progression (e.g., percentage declines in estimated glomerular filtration rate [eGFR]). We now report the effects of the total of two years of empagliflozin as study drug provided on risk of kidney disease progression and mortality outcomes during the active trial plus two years of post-trial observation.

## Methods

### Design

EMPA-KIDNEY was designed and conducted by the University of Oxford in collaboration with a Steering Committee (see Supplementary Appendix). The first and senior authors wrote the first-draft manuscript, vouch for the data, and made the decision to publish. The active trial’s rationale, double-blind placebo-controlled design and main results were reported previously.^3,7,8^ Relevant regulatory authorities and ethics committees for each participating center approved the trial and its post-trial follow-up. Adults with a race-adjusted kidney function formula (here CKD-EPI^9^) eGFR of ≥20 and <45 mL/min/1.73m^2^ (irrespective of level of albuminuria); or an eGFR of ≥45 and <90 mL/min1.73m^2^ with a urinary albumin-to-creatinine ratio (uACR) ≥200 mg/g at the active trial screening visit were eligible, provided they were prescribed a clinically appropriate dose of single renin-angiotensin system (RAS) inhibitor, when indicated and tolerated. Post-trial follow-up was an optional substudy conducted at 185 of the trial’s 241 centers (77%) in 7 of the original 8 countries. All willing surviving participants from participating centers were eligible for post-trial follow-up.

### Procedures

At the final active trial follow-up visit, all study drug was retrieved and local doctors informed about the trial’s conclusions. Investigators and participants remained blinded to treatment allocation, and no further study drug was provided to participants. Instead, local doctors were free to prescribe open-label SGLT2 inhibitors (where available and considered indicated) and were responsible for routine follow-up of kidney function as per local practice. Post-trial follow-up aimed to collect additional efficacy and cause-specific mortality outcome data. The main method of follow-up was medical record review supplemented with registry data in the UK and Malaysia. If medical records were unavailable, information was collected by contacting participants, a relative or carer, or local doctors. At 6 monthly reviews, details about vital status, current kidney replacement therapy status, latest local blood creatinine measurement, and any current use of relevant co-medication (limited to SGLT2 inhibitors, RAS inhibitors, and mineralocorticoid receptor antagonists) were collected directly into the trial’s custom-made IT system. Over 99% of reported ESKD was confirmed by local investigators, and reported deaths underwent central review and categorization by blinded clinician adjudicators following the same pre-specified definitions developed for the active trial.^3^

### Outcomes

The pre-specified primary post-trial assessment was the effect of allocation to empagliflozin during the original active trial on the time to the composite outcome of kidney disease progression or cardiovascular death occurring at any time during the entire follow-up period (i.e., the active trial and post-trial follow-up periods combined). Kidney disease progression was defined as a sustained ≥40% eGFR decline from randomization, ESKD, a sustained eGFR below 10 mL/min/1.73m^2^, or death from kidney failure.^3^ Confirmation of “sustained” required values on two consecutive eGFR measurements at least 30 days apart, or was assumed if it was the last eGFR value before death, withdrawal of consent or the end of a participant’s follow-up. In primary analyses, previously reported primary outcomes from the active trial period were carried over irrespective of later eGFR results collected during the post-trial period. As central samples were not collected during the post-trial follow-up period, all eGFR-based post-trial measurements were relative to the local eGFR measurement at baseline (see Supplementary Methods for more details).

The post-trial follow-up protocol pre-specified key secondary outcomes of kidney disease progression alone, and the composite of death from any cause or ESKD. The other secondary outcome was ESKD. Tertiary outcomes were death from any cause and, separately, death from cardiovascular and non-cardiovascular causes (the latter being the safety outcome for post-trial follow-up); and the primary outcome assessed by key subgroups of interest. These subgroups were by diabetes status, eGFR, uACR, and primary kidney disease at randomization (using categories pre-specified for the primary trial report^3^). Analyses explored the effect of empagliflozin on the primary and secondary outcomes by year, and eGFR in different follow-up windows.

### Statistical Analyses

The analyses were performed on the original full database developed and held by the University of Oxford. Pre-specified Cox proportional hazards regression models including adjustment for categorized baseline variables specified in the minimization algorithm (age, sex, prior diabetes, eGFR, uACR, and geographical region) were used to estimate hazard ratios (HR) and 95% CIs for empagliflozin versus placebo for time-to-event analyses.^10^ Surviving participants who did not enter post-trial follow-up (e.g., due to attending a non-participating site or being unwilling) were censored at the end of their active trial follow-up period. Kaplan-Meier estimates for the time to each of the primary and secondary outcomes were calculated. Absolute benefits per 1000 participants allocated empagliflozin were calculated from differences in Kaplan-Meier curves between allocated groups. The eGFR-based explorations used ANCOVA to estimate the baseline-adjusted absolute difference in mean eGFR at the last local measurement overall and for the four key subgroups, and mixed model repeated measures (MMRM) approaches to estimate mean eGFR at each follow-up time point throughout the entire follow-up period using only local laboratory measurements. Supplementary materials and the Data Analysis Plan provide further details of these methods. SAS software, version 9.4 (SAS Institute, Cary NY, USA) and R v4.3.2 were used for analyses.

## Results

### Recruitment and follow-up

Between May 2019 and April 2021, 6609 participants were randomized, entered the active trial period and were followed for a median of 2.0 (Q1-Q3 1.5-2.4) years. Of the 6253 participants not dead or withdrawn, 1362 (22%) did not provide consent for post-trial follow-up or were from sites that could not participate for logistical reasons (including all sites in Japan), leaving 4891 who entered the post-trial follow-up period. These participants were followed post-trial for a median of 2.0 (Q1-Q3 2.0-2.1) years. By the end of post-trial follow-up vital status was missing on or after 1 April 2024 for 86 of these 4891 participants (1.8%), and 7 (0.1%) withdrew consent during post-trial follow-up (Supplementary Appendix, Figure S1).

The subset of participants entering post-trial follow-up were broadly representative of the population of patients with CKD who are at risk of disease progression (Table S1), and baseline characteristics at randomization were broadly similar between the empagliflozin and placebo groups (Table 1). Mean (±SD) age of the post-trial participants at randomization was 63 (±14) years, 1664 (34%) were women, and 2784 (57%) did not have diabetes. The mean (±SD) eGFR was 36.9 (±14.1) mL/min/1.73m^2^ and median (Q1-Q3) uACR was 317 mg/g (44-1063), with 2393 (49%) with a uACR ≤300 mg/g. 3487 (71%) had a non-diabetic cause of CKD. Participants who entered post-trial follow-up were less likely to be Asian, slightly younger, had a slightly lower eGFR and uACR, and slightly higher risk of kidney failure when compared to survivors who did not enter post-trial follow-up (Table S2).

During the entire trial follow-up period, blinding to original study drug allocation was maintained in 6578 of the 6609 (>99%) participants. Average use of SGLT2 inhibitors during the active trial period was 90% in the empagliflozin group versus 2% in the placebo group. During post-trial follow-up, average use was similar between groups (43% vs. 40%, respectively; Table 2). Those participants who did not start an SGLT2 inhibitor during post-trial follow-up were more likely to be from Asia, less likely to have prior diabetes, had lower eGFR, notably higher kidney failure risk, and were less likely to be on a RAS inhibitor (Table S3). During post-trial follow-up, average use of RAS inhibitors declined over time but remained similar in both groups (68% vs. 68%) (Table S4).

### Primary and Secondary Outcomes

**Figure 1:**
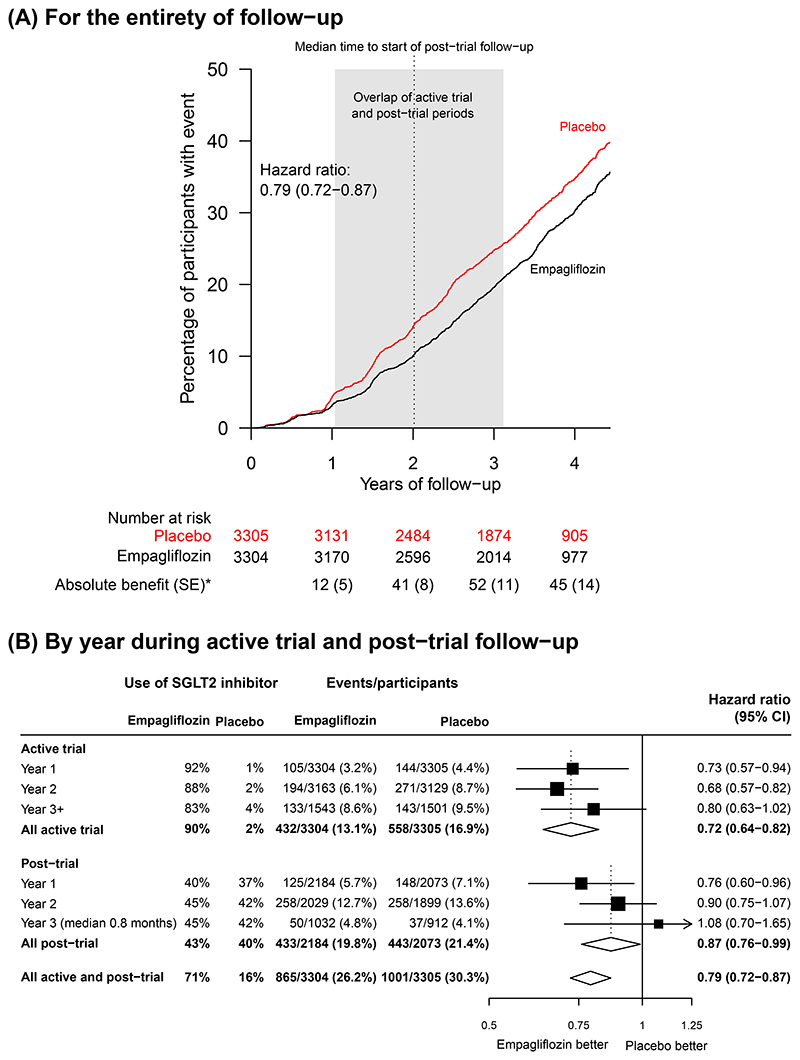
Effect of Allocation to Empagliflozin on Progression of Kidney Disease or Death from Cardiovascular Causes * Absolute difference in number of events per 1000 patients allocated to receive empagliflozin during the active trial period. Figure 1A provides the KM-plot for the primary outcome for the entire follow-up period (active and post-trial periods combined). The shaded area is wide as median follow-up of the active trial period was 2.0 years with range of 0.3-3.1 years. This wide range is a result of prolonged recruitment during the Covid-19 pandemic. By contrast, Figure 1B displays the annual ratios of the hazard rates in those originally allocated empagliflozin versus those originally allocated placebo separately for (a) the active trial, and (b) the post-trial period, during which time no participant took study drug but some were started on non-trial SGLT2i (not necessarily empagliflozin). Primary outcomes in the first 6 months post-trial 32/2184 (1.5%) vs. 49/2073 (2.4%), hazard ratio 0.60 (95% CI 0.38-0.93). Use of SGLT2 inhibitor defined in Table 2. Average use of SGLT2 inhibitors calculated using weights proportional to the total person years at risk in each year. Denominators are the number of participants still at risk of a first primary outcome at the start of the risk period.

During the entire follow-up period (active trial plus post-trial observation periods together), progression of kidney disease or death from cardiovascular causes occurred in 865 of 3304 patients (26.2%) in the empagliflozin group and in 1001 of 3305 (30.3%) in the placebo group (HR=0.79, 95%CI 0.72-0.87; Figure 1A). This comprised a 28% overall reduction in risk during the active trial period (0.72, 0.64-0.82; 990 outcomes) and a 13% reduction during the post-trial period (0.87, 0.76-0.99; 876 additional first primary outcomes). Much of the post-trial benefit on the primary outcome occurred early: hazard ratios for the first and second years of follow-up post-trial were 0.76 (0.60-0.96) and 0.90 (0.75-1.07) respectively, with the hazard ratio for first 6 months 0.60 (0.38-0.93) (Figure 1B). The sensitivity analyses yielded similar results (Figure S2 & Table S5).

The effect on the primary outcome during the entire follow-up period included a 21% reduction in the risk of the secondary outcomes of kidney disease progression (23.5% vs. 27.1%: 0.79, 0.72-0.87; Table 3 & Figure S3), and a 26% reduction in ESKD (9.0% vs. 11.3%: 0.74, 0.64-0.87; Table S5). During the post-trial period, the hazard ratios for kidney disease progression and ESKD were 0.89 (0.77-1.02) and 0.80 (0.66-0.98), respectively (Figure S3). During the entire follow-up period, there was a 19% reduction in the risk of the key secondary composite outcome of death from any cause or ESKD (16.9% vs. 19.6%: 0.81, 0.72-0.90), including a hazard ratio of 0.82 (0.70-0.96) for the post-trial period.

In absolute terms, compared with placebo there were 57 (SE14) fewer participants with a primary outcome per 1000 participants allocated to empagliflozin at the end of the active trial period, and 45 (14) fewer at the end of the entire follow-up period. This included 26 (8) and 25 (10) fewer participants with ESKD per 1000 participants allocated to empagliflozin, respectively. There were 25 (11) and 32 (12) fewer participants per 1000 allocated to empagliflozin with the composite outcome of death or ESKD at the end of active trial and entire follow-up periods (Table S6).

### Tertiary, Exploratory and Safety Outcomes

**Figure 2:**
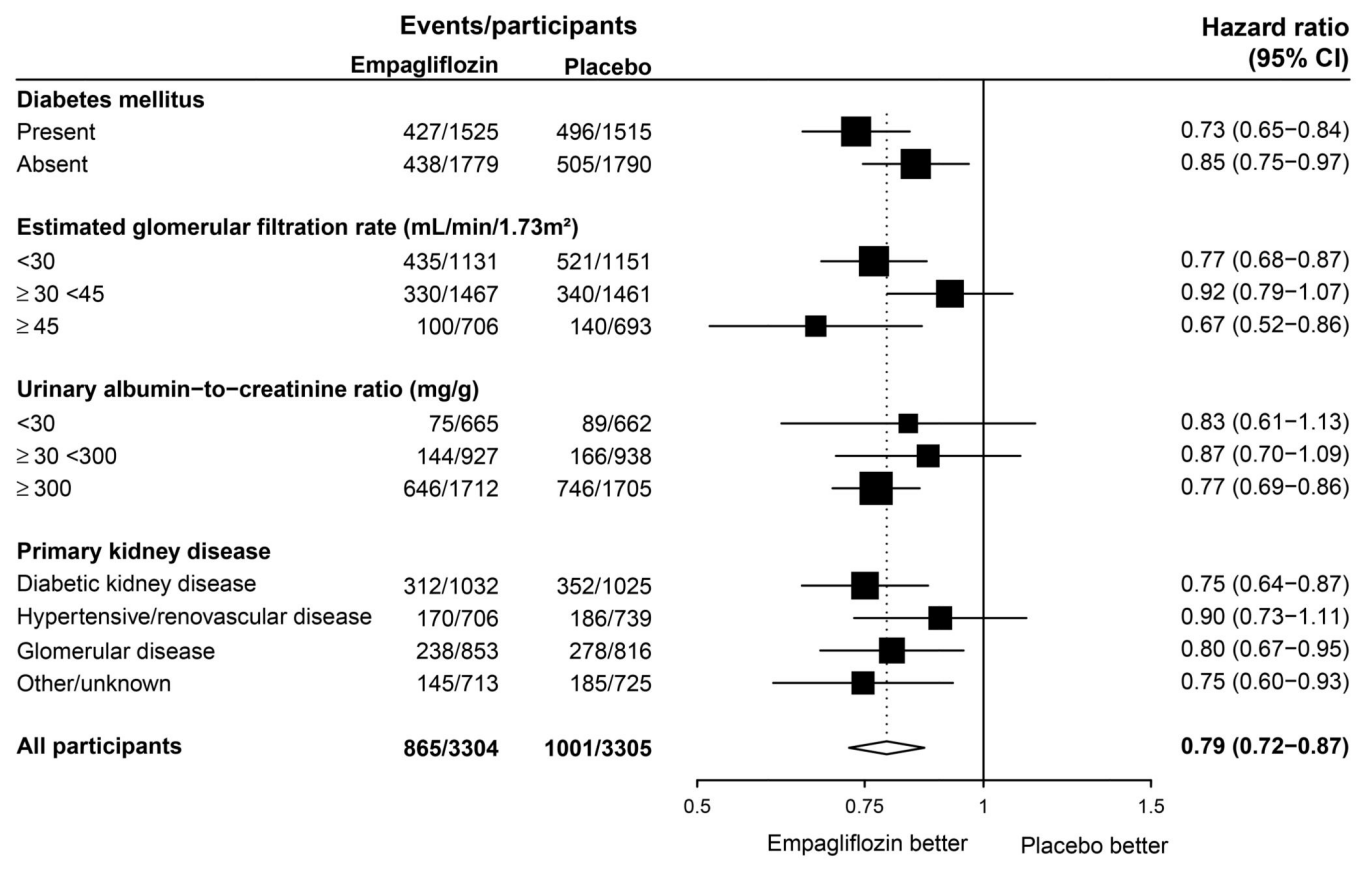
Effect of Allocation to Empagliflozin on Progression of Kidney Disease or Death from Cardiovascular Causes over the Entirety of Follow-up by Key Pre−specified Subgroups Entirety of follow-up is the active and post-trial periods combined. Presented analyses carry over the main results of the trial primary outcome from its active period.

The relative effects on the primary outcome were similar in subgroup analyses by baseline diabetes status, eGFR, uACR and primary cause of kidney disease (Figure 2). Findings were similar in post-hoc exploratory analyses assessing effects on kidney disease progression alone by key subgroups (Figure S4).

During the entire follow-up period, there was a reduction in the risk of death from a cardiovascular cause by 25% (3.8% vs. 4.9%: 0.75, 0.59-0.95) and no material effect on non-cardiovascular mortality (5.3% vs. 5.3%: 0.97, 0.79-1.20), meaning there were 301 (9.1%) vs. 336 (10.2%) deaths from any cause (0.86, 0.74-1.01; Table 3 and Figure S5).

Mean eGFR at last local measurement among those without ESKD was 31.4 (±0.2) mL/min/1.73m^2^ in the group originally allocated empagliflozin compared with 30.6 (±0.2) mL/min/1.73m^2^ in the placebo group, i.e., an absolute difference of 0.8 (95% CI 0.1-1.4) mL/min/1.73m^2^ (Table 3 and Figure S6). This absolute difference did not differ importantly in any of the key subgroups (Figure S7).

## Discussion

The results of the active period of EMPA-KIDNEY were previously reported; empagliflozin reduced the risk of the primary composite outcome of progression of kidney disease or cardiovascular death during 2 years of active trial treatment in its population of participants with a wide range of CKD etiologies, levels of kidney function and albuminuria, with no major safety concerns.^3^ We now report the results of two years of post-trial prospective observation in which patients and clinicians were blinded to participants’ original treatment allocation, and during which between-group use of SGLT2 inhibitors was similar. We found that there were residual cardiorenal benefits among patients allocated empagliflozin after study drug (empagliflozin or placebo) was discontinued. Mathematically, if there had been no off-treatment effect of empagliflozin post-trial (i.e. the hazard ratio was 1.0 after study drug stopped), absolute benefits would be observed to diminish from the end of the active trial period (see supplementary methods). Instead, we observed that absolute benefits both for the primary outcome and for the key secondary outcome of death or ESKD were initially maintained. In relative terms, the carry-over effect on the primary outcome was less than the effect of taking empagliflozin during the active trial period and appeared to last for up to 12 months, with most additional benefit exerted in the first 6 months after the active trial ended. Although not demonstrated directly, this suggests that to maximize the cardiorenal clinical benefits of SGLT2 inhibitors in CKD requires long-term treatment.

The mechanisms for any persisting effects of SGLT2 inhibitors still need to be elucidated. Preservation of nephron number during the active trial period might conceivably reduce hyperfiltration and risk of ESKD, and preservation of kidney function may have mediated some of the post-trial observed benefits on cardiovascular death.^11^ The acute eGFR dip with SGLT2 inhibition reversed within 4 weeks after discontinuation,^12,13^ so some of the observed post-trial benefit on eGFR components of kidney disease progression could result from reversal of the acute eGFR dip after cessation of study drug. However, that does not explain continuing benefits on ESKD.

Participants who did not start an SGLT2 inhibitor had twice the predicted risk of kidney failure of participants who started an SGLT2 inhibitor post-trial. This phenomenon may reflect some uncertainty about the efficacy of SGLT2 inhibition in those with more advanced CKD, and inertia in changes in practice owing to the time taken for regulatory/payer approvals. This prognostic imbalance implies, in particular, that any comparison of outcomes between participants allocated empagliflozin who started an SGLT2 inhibitor post-trial versus those allocated placebo who remained off SGLT2 inhibition post-trial will be confounded, and hence unreliable.

Current international guidance on use of SGLT2 inhibitors in CKD provide recommendations of different strengths for patients who were eligible for EMPA-KIDNEY based on different levels of albuminuria.^14^ The almost doubling of the number of first primary outcomes from 990 in the active trial’s report to 1866 after post-trial follow-up appears to help us to address uncertainties resulting from the active trial period.^3^ Benefits on the post-trial follow-up primary outcome, kidney disease progression, and difference in eGFR on last measurement were similar irrespective of level of albuminuria, as well as diabetes status, level of kidney function, and primary kidney diagnosis. This is consistent with effects on eGFR-slope from the active trial phase.^12,15^

Our trial was designed to ensure that findings would be widely generalizable. Other key strengths of this trial were its large size and broad eligibility criteria, high levels of adherence to study drug, the high volunteer rate for post-trial follow-up, and the almost complete follow-up.^3^ Post-trial follow-up addresses some of the limitations of the active trial, including its low number of cardiovascular deaths.

Limitations of the post-trial study include the exclusion of participants from Japan (where active trial treatment effects were similar to other regions^16^). Doing so did not bias presented hazard ratios. Additionally, post-trial follow-up relied on locally measured creatinine levels. We do not consider this a key limitation, as results of the active trial were very similar, irrespective of whether central or local creatinine values were used.^12^ The lack of additional data on hospitalizations - a deliberate decision to streamline post-trial follow-up as far as possible - prevented any assessment of any effects on hospitalization during the post-trial period.^3,17^

In summary, post-trial follow up of our trial participants quantified more completely the total effects of a short period of two years of empagliflozin treatment, including any carry-over effect after stopping study drug. In a broad range of patients with chronic kidney disease, empagliflozin continued to exert additional cardiorenal benefits for up to 12 months after it was discontinued.

Disclosure forms provided by the authors are available with the full text of this article at NEJM.org.

## Supplementary Material

supplement

## Figures and Tables

**Table 1:** Baseline Characteristics at Randomization for Participants Entering Post-trial Follow-up

	Empagliflozin (n=2472)	Placebo (n=2419)
**Demographics**		
Age at randomization (years)	63 (14)	63 (14)
Sex		
Men	1632 (66%)	1595 (66%)
Women	840 (34%)	824 (34%)
Race		
White	1552 (63%)	1503 (62%)
Black	91 (4%)	87 (4%)
Asian	791 (32%)	791 (33%)
Mixed	14 (1%)	6 (<1%)
Other	24 (1%)	32 (1%)
**Prior disease**		
Prior diabetes*	1087 (44%)	1020 (42%)
Prior cardiovascular disease§	639 (26%)	641 (26%)
**Clinical measurements**		
Systolic blood pressure (mmHg)	136.9 (18.3)	136.9 (18.3)
Diastolic blood pressure (mmHg)	78.6 (11.6)	78.6 (11.8)
Body mass index (kg/m^2^)	29.9 (6.6)	30.0 (6.7)
**Laboratory measurements**		
eGFR (mL/min/1.73m^2^)†		
Mean (SD)	36.9 (14.1)	36.9 (14.1)
<30	854 (35%)	857 (35%)
≥30 to <45	1128 (46%)	1082 (45%)
≥45	490 (20%)	480 (20%)
uACR (mg/g)†		
Geometric mean (approx. SE)	212 (9)	214 (9)
Median (Q1-Q3)	324 (44-1045)	313 (45-1079)
<30	515 (21%)	515 (21%)
≥30 to ≤300	686 (28%)	677 (28%)
>300	1271 (51%)	1227 (51%)
**Concomitant medication use**		
RAS inhibitor	2142 (87%)	2066 (85%)
Any diuretic	1028 (42%)	1052 (43%)
Any lipid-lowering medication	1638 (66%)	1582 (65%)
**Cause of kidney disease**		
Diabetic kidney disease	727 (29%)	677 (28%)
Hypertensive/renovascular disease	553 (22%)	572 (24%)
Glomerular disease	670 (27%)	636 (26%)
Other/unknown	522 (21%)	534 (22%)
**5 year predicted kidney failure risk (%)**		
Median (Q1-Q3)	10 (3-29)	10 (3-30)

Data are n (%), mean (SD), geometric mean (approx.. SE), or median (Q1-Q3). eGFR=estimated glomerular filtration rate. uACR=urinary albumin-to-creatinine ratio. RAS=renin-angiotensin system.

* Prior diabetes mellitus at randomization is defined as participant-reported history of diabetes of any type, use of glucose-lowering medication, or baseline HbA1c ≥48 mmol/mol at Randomization visit.

§ Prior cardiovascular disease defined as self-reported history of myocardial infarction, heart failure, stroke, transient ischemic attack, or peripheral arterial disease.

† Uses central measurement taken at the randomization visit, or most recent local laboratory result before randomization. Those with missing data for BMI (n=12) not presented in relevant rows.

**Table 2:** Use of SGLT2 Inhibitors by Time

	Empagliflozin	Placebo
**All participants**	N=3304	N=3305
Active trial period		
12 months	2920/3164 (92%)	21/3159 (1%)
24 months	1661/1884 (88%)	41/1875 (2%)
36 months	270/326 (83%)	12/323 (4%)
**Participants entering PTFU**	N=2472	N=2419
Active trial period		
12 months	2264/2423 (93%)	13/2363 (1%)
24 months	1319/1483 (89%)	30/1417 (2%)
36 months	254/297 (86%)	10/289 (3%)
Post-trial period		
12 months	885/2186 (40%)	804/2147 (37%)
24 months	1078/2376 (45%)	972/2312 (42%)

Active trial periods defined using 12, 24, and 36 months follow-up visit windows. Use in the active trial defined as a participant reporting taking ≥80% of allocated study drug or open-label SGLT2 inhibitor (denominators are those known to be alive in each period). Post-trial periods defined using information nearest to 12 and 24 months since completion of active trial follow-up. Post-trial open-label SGLT2 inhibitor use was ascertained from review of participant’s medical records, or where necessary, direct contact with participants (denominators are those who joined post-trial follow-up, had a follow-up in the period and were known to be alive in the relevant period). Use of open-label SGLT2 inhibitor in the empagliflozin group versus placebo group in the post-trial period after excluding participants on kidney replacement therapy in the relevant periods was 880/2032 (43%) vs 797/1960 (41%) at 12 months and 1069/2129 (50%) vs 961/2015 (48%) at 24 months.

**Table 3:** Effect of Allocation Empagliflozin on Primary, Secondary, Tertiary and Exploratory Estimated Glomerular Filtration Rate-based Outcomes over the Entirety of Follow-up --the Active Trial and Post-trial Follow-up Periods

	Empagliflozin (N=3304)		Placebo (N=3305)		
	Participants with event	No. of events per 100 patient years	Participants with event	No. of events per 100 patient years	Hazard ratio (95% CI)
**Primary outcome:**					
Progression of kidney disease or death from cardiovascular causes	865 (26.2%)	8.4	1001 (30.3%)	10.0	0.79 (0.72-0.87)
**Secondary outcomes:**					
Kidney disease progression	778 (23.5%)	7.5	897 (27.1%)	9.0	0.79 (0.72-0.87)
Death from any cause or ESKD	559 (16.9%)	5.1	648 (19.6%)	6.1	0.81 (0.72-0.90)
ESKD	296 (9.0%)	2.7	372 (11.3%)	3.5	0.74 (0.64-0.87)
**Tertiary outcomes:**					
Death from any cause	301 (9.1%)	2.7	336 (10.2%)	3.0	0.86 (0.74-1.01)
Death from cardiovascular cause	126 (3.8%)	1.1	162 (4.9%)	1.5	0.75 (0.59-0.95)
Death from non-cardiovascular cause	175 (5.3%)	1.5	174 (5.3%)	1.6	0.97 (0.79-1.20)
	**Participants with measurement**	**Mean eGFR at last measurement**	**Participants with measurement**	**Mean eGFR at last measurement**	**Absolute difference (95% CI)**
Mean eGFR at last local measurement, mL/min/1.73m^2^*	3295 (>99%)	31.4 (0.2)	3295 (>99%)	30.6 (0.2)	0.8 (0.1,1.4)

ESKD=end-stage kidney disease. eGFR=estimated glomerular filtration rate. All analyses include all participants for the active trial period preserving the active trial results.

* Last local measurement defined as the last local creatinine value recorded prior to death, ESKD (i.e. date of commencement of maintenance dialysis or receipt of a kidney transplant), withdrawal of consent, or end of follow-up. Absolute difference estimated using ANCOVA. There were no serious adverse events attributed to study drug during post-trial follow-up in either group.
