# Supplementary material for: Long-Term Effects of Empagliflozin in Patients with Chronic Kidney Disease
Source: N Engl J Med. Author manuscript; Available in PMC 2024 Oct 28. (PMC7616743; doi:10.1056/NEJMoa2409183)
Supplement: supplement [file EMS199024-supplement-supplement.pdf]

# Long-Term Effects of Empagliflozin in Patients with Chronic Kidney Disease

The EMPA-KIDNEY Collaborative Group

Supplementary Appendix

**Members of the EMPA-KIDNEY Collaborative Group** ........ 3
**Membership of the Executive Committee, Steering Committee and Independent Data Monitoring Committee** ........ 3
**Central and Regional Coordination** ........ 3
**List of Collaborators, by Site** ........ 4
**Supplementary Methods** ........ 12
**EMPA-KIDNEY definitions of end-stage kidney disease and approaches to verification of deaths using medical notes (clinical adjudication)** ........ 13
**Cardiovascular death definitions** ........ 15
Myocardial infarction death ........ 15
Other coronary heart disease death ........ 15
Other cardiac disease death (including sudden cardiac death) ........ 15
Stroke death ........ 16
Other cardiovascular death ........ 16
Unexplained death ........ 17
**Non-cardiovascular death definitions** ........ 17
Renal death ........ 17
Infection death ........ 17
Cancer death ........ 17
Other specific medical causes of death ........ 18
Non-medical death ........ 18
**Supplementary Statistical Methods** ........ 19
Time-to-first event analyses ........ 19
Estimates of absolute benefits ........ 19
Mixed model repeated measures (MMRM) approach ........ 20
**Supplementary Figures** ........ 21
**Figure S1.** CONSORT participant flowchart for active and post−trial follow-up periods (PTFU) ........ 22
**Figure S2:** Effect of allocation to empagliflozin on progression of kidney disease or death from cardiovascular causes, restricted to sites that contributed to post-trial follow-up (sensitivity analysis) ........ 23
**Figure S3:** Effect of allocation to empagliflozin on secondary outcomes over the entirety of follow-up ........ 24

**Figure S4:** Effect of allocation to empagliflozin on progression of kidney disease ............ 25

**Figure S5:** Effect of allocation to empagliflozin on cause‒specific mortality over the entirety of follow-up .......... 26

**Figure S6:** Effect of allocation to empagliflozin on estimated glomerular filtration rate over the entirety of follow‒up ............ 27

**Figure S7:** Effect of allocation to empagliflozin on absolute difference in mean estimated glomerular filtration rate at last local measurement by key pre‒specified subgroups ............ 28

**Supplementary Tables** ............ 29

**Table S1:** Representativeness of study participants ............ 30

**Table S2:** Baseline characteristics at randomization for participants in post-trial follow-up (PTFU) and those not in PTFU ............ 31

**Table S3:** Baseline characteristics at the last follow-up during the active trial treatment phase for participants entering post-trial follow-up (PTFU) and those not in PTFU and by use of SGLT2 inhibitors (SGLT2i) at the end of PTFU ............ 32

**Table S4:** Use of RAS inhibitors and mineralocorticoid receptor antagonists over time ............ 34

**Table S5:** Effect of allocation to empagliflozin on components of primary outcome over the entirety of follow-up (i.e. combining active trial and post-trial follow-up periods) ............ 35

**Table S6:** Absolute benefits of allocation to empagliflozin on primary and secondary outcomes over the entirety of follow-up ............ 36

# Members of the EMPA-KIDNEY Collaborative Group

## Membership of the Executive Committee, Steering Committee and Independent Data Monitoring Committee

*Executive Committee*
Colin Baigent (co-Chair), Martin J. Landray (co-Chair), Christoph Wanner (Deputy Chair), William G. Herrington (Chief Investigator), Richard Haynes (co-Principal Investigator), Jennifer B. Green, Sibylle J. Hauske*, Martina Brueckmann*, Mark Hopley*
(Previous members: Maximillian von-Eynatten* & Jyothis George*)

*Steering Committee*
Executive Committee members plus National Representatives: Susanne Brenner (Germany); Alfred K. Cheung (United States); David Preiss (United Kingdom); Zhi-Hong Liu, Jing Li (China); Laiseong Hooi, Wen Liu (Malaysia); Takashi Kadowaki, Masaomi Nangaku (Japan); Adeera Levin, David Cherney (Canada); Roberto Pontremoli, Aldo P. Maggioni (Italy); plus statistician members: Natalie Staplin, Jonathan Emberson, Stefan Hantel*; plus other expert members: Shinya Goto, Rajat Deo, Katherine R. Tuttle. Non-voting members: Michael Hill, Parminder Judge, Kaitlin J. Mayne, Sarah Y.A. Ng, Xavier Rossello, Emily Sammons, Doreen Zhu

* denotes a Boehringer Ingelheim employee

*Independent Data Monitoring Committee*
Peter Sandercock (Chair), Rudolf Bilous, Charles Herzog, Paul Whelton, Janet Wittes, Derrick Bennett (non-voting statistician)

## Central and Regional Coordination

## Central Coordinating Office

***Administration***: Patricia Achiri, Chrissie Ambrose, Cristina Badin, Jill Barton, Richard Brown, Andy Burke, Sebastian Butler, Rejive Dayanandan, Pia Donaldson, Robert Dykas, Lucy Fletcher, Kate Frederick, Hannah Kingston, Mo Gray, Emily Harding, Akiko Hashimoto, Lyn Howie, Susan Hurley, Ryonfa Lee, Nik Luker, Kevin Murphy, Mariko Nakahara, John Nolan, Michelle Nunn, Sorcha Mulligan, Akiko Omata, Sandra Pickworth, YanRu Qiao, Shraddha Shah, Karen Taylor, Alison Timadjer, Monique Willett, Liz Wincott, Qin Yan, Hui Yu, Nichola Jones, Bridget Henderson, Genna Bobby; ***Clinical***: Louise Bowman, Fang Chen, Robert Clarke, Michelle Goonasekera, Richard Haynes, William G. Herrington, Parminder Judge, Waseem Karsan, Marion Mafham, Kaitlin J. Mayne, Sarah Y. A. Ng, David Preiss, Christina Reith, Emily Sammons, Mohammed Zayed, Doreen Zhu, Nikita Agrawal, Ryoki Arimoto; ***Data Analysis:*** Ritva Ellison, Rowan Moys, Will Stevens, Kevin Verdel, Karl Wallendszus; ***Finance:*** Chris Bowler, Anna Brewer, Andy Measor; ***IT Validation:*** Guanguo Cui, Charles Daniels, Angela Field, Bob Goodenough, Ashley Lawson, Youcef Mostefai, Dheeptha Radhakrishnan, Samee Syed, Shuang Xia; Laboratory: Ruth Adewuyi-Dalton, Thomas Arnold, Anne-Marie Beneat, Anoushka Bhatt, Chloe Bird, Andrew Breach, Laura Brown, Mark Caple, Tatyana Chavagnon, Karen Chung, Sarah Clark, Luminita Condurache, Katarzyna Eichstadt, Marta Espino Obrero, Scarlett Forest, Helen French, Nick Goodwin, Andrew Gordon, Joanne Gordon, Cat Guest, Tina Harding, Michael Hill, Michal Hozak, Matthew Lacey, David MacLean, Louise Messinger, Stewart Moffat, Martin Radley, Claire Shenton, Sarah Tipper, Jon Tyler, Lesley Weaving, James Wheeler, Elissa Williams, Tim Williams, Hamish Woodhouse; ***Monitoring:*** Angela Chamberlain, Jo Chambers, Joanne Davies, Denise Donaldson, Pati Faria-Shayler, Denise Fleming-Brown, Jennifer Ingell, Carol Knott, Anna Liew, Helen Lochhead, Juliette Meek, Isabel Rodriguez-Bachiller, Andrea Wilson, Patrick Zettergren, Meera Mistry; ***Programming:*** Rach AitSadi, Ian Barton, Alex Baxter, Yonghong Bu, Lukasz Danel, Sonja Grotjahn, Rijo Kurien, Michael Lay, Archie Maskill, Aleksandra Murawska, Rachel Raff, Allen Young; ***Principal Investigators:*** Colin Baigent, Richard Haynes,

William G. Herrington, Martin J. Landray, David Preiss; ***Statistics:*** Jonathan Emberson, Rebecca Sardell, Natalie Staplin

**Regional Coordinating Centres**

**Germany (Universitätsklinikum Würzburg):** Christoph Wanner, Susanne Brenner, Vladimir Cejka, Marcela Fajardo-Moser, Christian Hartner, Doris Poehler, Janina Renner, Franziska Scheidemantel, Sharang Ghavampour

**United States (Duke Clinical Research Institute [DCRI])**: Jennifer B. Green, Miya Bryant, Anita Hepditch, Cassandra Johnson, Erin Latore, Yolanda Miller, Lauren Price, Merilee Whalen, Ashleigh Wheeler

**UK (Clinical Trial Service Unit and Epidemiological Studies Unit [CTSU], University of Oxford)**: Richard Haynes, David Preiss, Cristina Badin, Jo Chambers, Joanne Davies, Denise Donaldson, Mo Gray, Emily Harding, Jenny Ingell, Yanru Qiao, Shraddha Shah, Andrea Wilson, Patrick Zettergren

**China (National Center for Cardiovascular Disease, Fuwai Hospital & National Clinical Research Center of Kidney Diseases, Jinling Hospital, Nanjing University School of Medicine):** Zhi-Hong Liu, Jing Li, Yu An, Yinghua Chen, Peiling Chen, Hao Dai, Hong Du, Fang Feng, Qing Guo, Libo Hou, Wuhanbilige Hundei, Binbin Jin, Yan Li, Jiamin Liu, Xia Song, Yanping Wang, Yanwu Yu, Ning Zhang, Lingshan Zhao, Hui Zhong, Yi Yang, Ying Sun

**Malaysia (Klinsel SDN BHD)**: Cheng Beng Goh, Ye Mun Low, Soon Yi Sor, Farah Hanis Zulkipli, Sarojini Sivanandam, Nurusyifaa' Nadhirah Mohd Shahfari

**Japan (Parexel):** Natsuki Arai, Ai Fukasawa, Mizue Furukawa, Keisuke Habuki, Shoko Hayashi, Wakako Isari, Saki Kanegae, Maria Kawai, Reiki Kobayashi, Takako Kuramae, Chika Kuribayashi, Sawako Maeno, Satoshi Masumoto, Tomoko Morisaki, Minoru Oda, Kazue Sawada, Kenta Sugamori, Ayana Tatsuzawa, Aiko Tomita, Kazuyuki Yuasa, Hiroko Inazawa

**Canada (Providence Health Care, Vancouver):** Adeera Levin, Amanda Axler, Kerri Gallo

**Italy (ANMCO Research Center):** Aldo P. Maggioni, Ester Baldini, Barbara Bartolomei Mecatti, Francesca Bianchini, Martina Ceseri, Laura Cipressa, Gianna Fabbri, Andrea Lorimer, Donata Lucci

**List of Collaborators, by Site**

**Germany:** Universitatsklinikum Wurzburg: Christoph Wanner, Susanne Brenner, Vladimir Cejka, Sharang Ghavampour, Anja Knoppe, Tereza Cairns; Zentrum fuer Nieren-, Hochdruck- und Stoffwechselerkrankungen Hannover: Hans Schmidt-Gurtler, Hubert Dumann, Sybille Merscher, Margret Patecki, Georg Rainer Schlieper, Anke Torp, Bianca Weber, Maja Zietz; Nephrologisches Zentrum Villingen-Schwenningen: Bernd Hohenstein, Urs Benck, Diliana Draganova, Thomas Weinreich, Lothar Wolf, Jasmine Gaidu, Hanna Reiner, Mandy Visnjic; Nierenzentrum Freiburg: Daniel Steffl, Marie Breitenfeldt, Annette Kraemer-Guth, Christine Braun, Simone Hagge; Dialysezentrum Heilbronn: Michael Schomig, Stephan Matthias, Dominik Stoffler, Beate Schumacher; Klinikum der Universitat Munchen: Thomas Sitter, Louise Fuessl, Julia Krappe, Jerome Loutan, Volker Vielhauer, Luciano Andriaccio, Magdalena Maurer, Sybille Spies; ClinPhenomics GmbH Co. KG: Bernhard Winkelmann, Martin Dursch, Linda Seifert, Linda Tenbusch, Gudrun Schneckenburger, Tina Geinitz, Kerstin Michalek, Simon Steininger, Julia Mueller; Universitatsmedizin Mainz: Julia Weinmann-Menke, Simone Boedecker, Wiebke Kaluza-Schilling, Daniel Kraus, Carina Krieger, Margit Schmude, Anne Schreiber, Ewelina Eckrich; Herz- und Diabeteszentrum Nordrhein-Westfalen: Diethelm Tschope, Abdulwahab Arbi, Young Lee-Barkey, Bernd Stratmann, Natalie Prib, Sina Rolfsmeier, Irina Schneider; Universitatsklinikum Dusseldorf: Lars Rump, Johannes Stegbauer, Christine Pötz, Mara Schemmelmann, Claudia Schmidt, Sinje Landmann; Nephrocare Mettmann -

Standort Velbert: Michael Koch, Sendogan Aker, Annika Küpper, Manuela Martin; Diaverum MVZ Potsdam: Thiemo Pfab, Christian Albert, Michael Haase, Barbara Zander, Claudia Schneider-Danwitz; Praxis fur Dialyse und Nierenkrankheiten - Arztezentrum Helle Mitte: Wolfgang Seeger, Wolf-Adam Seeger, Britta Zemann; Klinikum Bielefeld: Christoph Stellbrink, Kristin Marx, Ekaterina Stellbrink, Britta Brettschneider, Stephanie Watson, Marion Iselt; Studienzentrum Aschaffenburg: Gerhard Klausmann, Inga-Nadine Kummer, Auguste Kutschat, Simone Streitenberger; Universitatsklinikum Halle: Matthias Girndt, Silke Markau, Ina Girakossyan, Claudia Hanf; Klinikum St. Georg Leipzig: Joachim Beige, Ralph Wendt, Ulrike Schmidt, Birgit Labitzke, Leoni Leistner; Studienzentrum Nephrologie Nurnberg-Langwasser: Andreas Schneider, Roland Veelken, Claudia Donhauser, Auguste Kutschat; UBAG fur Nephrologie und Dialyse Neckarsulm: Luis Becker, Nexhat Miftari, Ricarda Wolfling, Sarah Morlok; Universitatsklinikum Dresden: Christian Hugo, Alexander Paliege, Jens Passauer, Julian Stumpf, Annegret Fleischer, Kerstin Haaser; Universitatsklinikum Mannheim: Bernhard Kraemer, Jan Jochims, Bernd Kruger, Claudia Foellinger, Anastassiya Reisler; Nierenzentrum Wiesbaden: Frank Strutz, Stefan Haack, Ursula Hohenstatt; Universitatsklinikum Jena: Martin Busch, Konstantin Herfurth, Gunter Wolf, Rainer Paul, Andy Steiner; Studienzentrum fur Nieren- und Hochdruckerkrankungen Hannover: Hermann Haller, Jessica Kaufeld, Jan Menne, Elisabeth Bahlmann-Kroll, Angela Bergner, Kai Schmidt-Ott; Universitatsklinikum Augsburg: Horst Weihprecht, Aydin Er, Florian Sonntag, Elif Turan, Michael Wittmann, Franziska Klauser, Eva Voigt, Julia Gatzschmann, Franziska Thieme; Nephrologisches Zentrum Gottingen: Volker Schettler, Egbert Schulz, Madlen Rohnstock, Elke Schettler; Universitaetsklinik Ulm: Bernd Schroppel, Rene van Erp, Martin Kachele, Ulla Ludwig, Lena Schulte-Kemna, Waltraud Kmietschak, Elke Preiss, Martina Ruocco; AGAPLESION Markus-Krankenhaus: Gunnar Heine, Martin Brzoska, Sebastian Gabel, Christina Büttner, Asma Sabarai, Christina Buttner; Universitatsklinikum Regensburg: Bernhard Banas, Tobias Bergler, Yvonne Ehrl, Franz Putz, Antonia Schuster, Stefanie Kuhn, Torsten Schramm; DaVita Viersen - Nettetal: Stefan Degenhardt, Gerhard Schmidt, Lea Weiland, Ulrike GiebelnHudnell; Klinikum Braunschweig: Jan Kielstein, Gabriele Eden, Brigitte Fuchs, Gina Morig, Manuela Winkler, Christina Engel; Nephrocare Mettmann: Michael Koch, Sendogan Aker, Annika Küpper, Manuela Martin; Vivantes Klinikum Neukolln: Harald Darius, Charalampos Kriatselis, Carl- Philipp Roesch; Astrid Maselli, Robert-Bosch-Krankenhaus Stuttgart: Dominik Alscher, Markus Ketteler, Moritz Schanz, Severin Schricker, Bianka Rettenmaier, Andrea Schwab, Fateme Rahimi

**United States:** Clinical Advancement Center: Pablo Pergola, Irene Leal, Melissa Cagle, Anna Romo, Anthony Torres, NataliaCabrera; Seacoast Kidney and Hypertension Specialists: Sucharit Joshi, Kulli Barrett, Alexis Africano, Vicki Dodds, Dorleena Gowen, Ashlee Morris, Stacey Perry; Total Research Group, LLC: Juan Fernandez, Guillermo Jimenez, Ricardo Viera, Kendaling Bruce, Ryan Barrios, Maylin Garcia, Kerelyn Garcia, Iradis Leal; Nephrology Consultants, LLC: David Tietjen, David Bains, Carlo Castillo, Genielle Brewer, Justin Davis, Natalie Freking, Brittany Golson, Sally Ham, Jesslyn Roesch; Sumter Medical Specialists: Pusadee Suchinda, Shameem Beigh, Usah Lilavivat, Joyce Bilton, Kim Bocchicchia, Heidi Griswold; Yale University: Jeffrey Turner, Neera Dahl, Aldo Peixoto, Yasemin Kavak, Lauren Liberti, Hari Nair, Nicolas Page, Stephanie Rosenberg, Kathryn Simmons; Northwestern University: Tamara Isakova, Rebecca Frazier, Rupal Mehta, Anand Srivastava, Patrick Fox, Jonathan Hecktman, Alexander Hodakowski, Carlos Martinez, Rachel Phillips, Alexis Stevenson, Marija Zimkute, Reed Jaworski; University of Kansas Medical Center: Reem Mustafa, Kyle Jansson, Cassandra Kimber, Jason Stubbs, Ahmad Tuffaha, Sri Yarlagadda, Debbie Griffin, Elisabeth Laundy, Zhuo Tang, Casey Tan, Abigayle Joyce; Providence Sacred Heart Medical Center and Childrens Hospital: Radica Alicic, Katherine R. Tuttle, Ann Cooper, Lisa Davis; East Coast Research Institute: Ashwini Gore, Rebecca Goldfaden, Leslie Harvill, Lisa Hichkad, Barry Johns, Thomas Jones, Kayla Merritt, Jennifer Sheldon, Jennifer Stanfield, Lindsay Alexander, Kaitlyn Preston, Lindsey Wood; Monument Health: Rajesh Pradhan, Roger DeRaad, Kelli McIntosh, Louis Raymond, Michael Shepperd, Susan McLaughlin, Mary Seifert, Andrew Shepherd; Mountain Kidney & Hypertension Associates: Joseph Aiello, William Durham, Laurie Loudermilk, John Manley, Sabrina Burnette, Stephanie Evans, Tara Johnson; Texas Institute for Kidney and Endocrine Disorders: Lance Sloan, Judy Ann Acosta, Stacy Gillham, Katia Sloan, SueAnn Squyres; Wake Forest University Health Sciences: Michael Rocco, Amret Hawfield, Ben Bagwell, Lauren Richmond; Chase Medical Research: Joseph Soufer, Subha Clarke, Amanda Aliu, Kristine Calabrese, Amanda Davis, Veronica Poma, Tracy

Spinola; East Coast Institute for Research LLC: James Magee, Ricardo Silva, Rushab Choksi, Lorraine Dajani, John Evans, Anil George, Rebecca Goldfaden, Prasanth Krish, Gerard Martins, Mae Sheikh-Ali, David Sutton, Freda Driver, Abraham Hanburry, Laura Hume, Amber Hurst, Matthew Taddeo, Marla Turner, Veronica Yousif; University of Utah Health Sciences: Srinivasan Beddhu, Laith Al-Rabadi, Nikita Abraham, Amalia Caamano, Judy Carle, Victoria Gonce, Kaitlyn Staylor, Na Zhou; University of Texas Health Science Center at San Antonio: Shweta Bansal, Manoj Bhattarai, Kumar Sharma, Subrata Debnath, Aliseiya Garza, Chakradhar Velagapudi; Academy of Diabetes, Thyroid, and Endocrine, PA: Sergio Rovner, Javier Almeida, Pablo Casares, Verlaine Stewart-Ray, Rene Almaraz, Renata Dayrell, Ana Moncada, Ricardo Pulido, Roxana Rodriquez; East Coast Institute for Research: James Magee, Wasim Deeb, Kathryn DeGoursey, Rodel Gloria, Trevor Greene, Robert Miller, Edward Pereira, Miguel Roura, Mae Sheikh-Ali, David Sutton, Debbie Domingo, Sasha Dorestin, William Hodge, Cathy Jackson, Deborah Lund, Katrina Taylor; Aventiv Research: Kenneth Boren, Brittany Cleveland, Sandra Gaiser, Mandeep Sahani, Logan Aldrich, Exodus Edmerson, Edmond Limon, Cole Valletta, Patricia Vasquez, Amanda Harrington, Haley Edwards, Jennifer Green; St. Clair Nephrology Research: Christopher Provenzano, Navkiranjot Brar, Heather Henderson, Bellovich Keith, Qur Khai, Quresh Khairullah, Gail Makos, Joel Topf, Sherry Gasko, Rosemarie Henschel, Kaitlin Knapp, Teresa Kozlowski, Paula LaFleur, Ashwathy Varughese; Kaiser Permanente San Diego: Hui Xue, Patricia Wu, Olga Arechiga, Shan Darbeau, Michael Fechter, Stephanie Martinez, Katherine Klein, Eva Rodriguez; Hanson Clinical Research Center: Lenita Hanson, Nyla Cooper, Arelis Madera, Jay Cadorna, Rita Sheridan, Helen Sparks; Saint Elizabeth Healthcare: Bradley Eilerman, Susanne Bodine, Wael Eid, Rebecca Flora, Amber Avery, Cashmere Hardy; Thomas Jefferson/ARIA Health Northeast Endocrine Metabolic Associates: Mihaela Biscoveanu, Steven Nagelberg, Tracey Cummins; Emory University: Frederic Rahbari-Oskoui, Anju Oommen, Zohreh Forghani, Stacie Hitchcock, Darya Hosein, Diane Watkins; East Coast Institute Research, LLC: Minesh Patel, Anthony Lambert, Elizabeth Newman, Autumn Wood, Tammy Ross, Stephany Topping; Kidney Care and Transplant Services of New England: Jeffrey Mulhern, Lorna Murphy, Ann Vasseur; Brookview Hills Research Associates LLC: Gregory Greenwood, Alexander Hadley, Denise Laurienti, Christopher Marshall, Nicholas McLean, Scott Satko, Brandy Caudill, Jacob Maris, Janice Rogers, Cindy Vanhoy; Cleveland Clinic: George Thomas, Georges Nakhoul, John O'Toole, Jonathan Taliercio, Leslie Cooperman, Marina Markovic, Barbara Tucky; Salem V.A. Medical Center: Devasmita Dev, Alia Hasan, Hima Yalamanchili, Namita Jain, Lesley McNeil, Eric Wines; Medstar Health Research Institute: Jean Park, Adline Ghazi, Mia Hamm, Tejas Patel; University of North Carolina Hospital: Amy Mottl, Emily Chang, Vimal Derebail, Emmie Cole, Anne Froment, Sara Kelley, Jordan Osmond Foster; Olive View - UCLA Medical Center: Vahid Mahabadi, Golriz Jafari, Anita Kamarzarian, Wendy Arriaga, Daisy Arteaga, Rosario Machicado, Genesis Naverrete; P&I Clinical Research, LLC: Prashant Kumar, Imran Nazeer, Karina Urquia, Tammi Glider, Vickie Jones, Savannah Rucker, Jennifer Wiley, Tammy Rider; Pioneer Research Solutions: Rahul Pandey, Jesus Arroyo, Harish Pariani, Mohammad Ahmad, Shahin Mozaffari, Erika Perez, Andres Miranda; Los Angeles Biomedical Research Institute at Harbor-UCLA Medical Center: Matthew Budoff, Sion Roy, Divya Birudaraju, Ahmed Ghanem, Sajad Hamal; Research Institute of Dallas: Stephen Aronoff, Elisa Joye Petr, Richard Sachson, Jaime Wiebel, Sana Akram, Laurie Jones, Curtis Knight; Maurie Tarlac, Idara Ukpong, Kim Quiroga; Renal Disease Research Institute: Shahbaz Ahmed, Harold Szerlip, Akinwande Akinfolarin, Ankit Mehta, Shana Camp, Cindy Castro, Zanaida Cooper, Jessica Terry; Clinical Research Consultants: Ahmed Awad, Bhavya Kothapalli, Ryan Lustig, Serine Alfaress, Hyder Jasim, Mary Parrigon; Lexington V.A. Health Care System: Dennis Karounos, Sadiq Ahmed, Maggie Berry, Ruth Oremus; VA Southern Nevada Healthcare System: Carlos Hernandez-Cassis, Elias Ugwu, Nazia Junejo, Nancy Suazo, Todd Clark, Rosalinda Cruz; University of Florida Health: Mark Segal, Amir Kazory, Sherry Brown, Tristan Daniels, Sofia Dayi, Renee Hogan, Kathy McCray, Jennifer Stickley; University Hospitals Cleveland Medical Center: Mahboob Rahman, Mirela Dobre, Lavinia Negrea, Aparna Padiyar, Nishigandha Pradhan, Arash Rashidi, Nagaraju Sarabu, Vicki Donley, Tricia Young, Elizabeth DeCaro; Midland Florida Clinical Research Center: Godson Oguchi, Judepatricks Onyema, Kahla Damianik, Jack Dienes, Judith Plummer-Morgan, Marilyn Roman, Mauver Skipper, Stacey-Ann Villaruel, Krystle Williams, Svetlana Shilo, Numaliz Chokr; Cedar Crosse Research Center: Danny Sugimoto, Jeffrey Dugas, Ismeal Ahmed, Jamie Bhairoo, Dolores Rijos, Huzaifa Salim, Kaleena Urquidi

**UK:** Oxford University Hospitals: Richard Haynes, William G. Herrington, Doreen Zhu, Madita Gavrila, Kathryn Lafferty, Ria Rabara, Sally Ruse, Maria Weetman; Southmead Hospital, Bristol: James Bushnell, Albert Power, Alison Jenkins, Stefanie Jones, Amanda Scott; Nottingham City Hospital: Cath Byrne, Mark Jesky, Alison Cowley, Emma McHaffie, Holly Waterfall, Neha Bhalla; Dorset County Hospital: Jo Taylor, Laura Bough, Thomas Phillips, Barbara Winter-Goodwin, Keegan Lee; King's College Hospital, London: Sui Phin Kon, Iain MacDougall, Eirini Lioudaki, Sapna Shah, Claire Sharpe, Francisco Aguilar, Abegail Hernandez Pena, Conception Pugay, Amelia Te, Tony Johny, Philip Francisco; Queen Elizabeth Hospital Birmingham: Hugh Finn, Wasim Hanif, Samiul Mostafa, Alice Aitken, Katharine Draxlbauer, Evelina Grobovaite, Jennifer Kearney, Theresa McCarthy, Faye Moore, Christianah Morakinyo, Sephora Thorpe; Royal Cornwall Hospital: Giorgio Gentile, Duncan Browne, Palanichamy Chellamuthu, Tabinda Dugal, Terri Chant, Laura Jones, Emily Laity, Megan Miners, James Muir, Elizabeth Swanson; Imperial College Healthcare NHS Trust: Andrew Frankel, James Tomlinson, Marlon Alegata, Rashid Almasarwah, Anthoula Apostolidi, Maria Vourvou, Thomas Walters; Royal Derby Hospital: Maarten Taal, Hari Dukka, Nitin Kolhe, Carly McDonald, Kelly White; The Queen Elizabeth Hospital, King's Lynn: Shiva Ugni, Smita Gunda, Rotimi Oluyombo, Vicki Brindle, Ping Coutts, Tracy Fuller, Evelyn Nadar; Princess Royal Hospital, Telford: Suresh Ramadoss, Denise Donaldson, Nichola Motherwell, Susannah Pajak, Louise Tonks, Mandy Beekes; Hull Royal Infirmary: Sunil Bhandari, Richard Bodington, Adil Hazara, Dominic Fellowes; University Hospital Aintree: Christopher Wong, Christopher Goldsmith, Sherald Barnes, Ann Bennett, Claire Burston, Samantha Hope, Nicola Hunt, Lini Kurian; UHNM Royal Stoke University Hospital: Richard Fish, Daniela Farrugia, Judy Lee, Emma Sadler, Hannah Turner; Belfast City Hospital: Christopher Hill, Henry Brown, Agnes Masengu, Peter Maxwell, Nina Bleakley, Hugh Murtagh; West Suffolk NHS Foundation Trust: William Petchey, Vivian Yiu, Joanne Kellett, Angharad Williams, Veronica Mendez Morro; Royal Devon and Exeter Hospital: Helen Clarke, Victoria Carnall, Sarah Benyon, Caroline Blake, Stephanie Estcourt, Jane Piper, Gigee Joseph; Daisy Hill Hospital: Neal Morgan, Carolyn Hutchinson, Teresa McKinley; Ulster Hospital, Dundonald: Alastair Woodman, Judi Graham, Niall Leonard, John Smyth, Vicki Adell, Samantha Hagan; Royal Free London NHS Foundation Trust: Ben Caplin, Amin Oomatia, Eleanor Damian, Toluleyi Sobande, Phil Gardiner; Kent & Canterbury Hospital: Tim Doulton, Michael Delaney, Mahmoud Montasser, Jenny Hansen, David Loader, Angela Moon, Frances Morris; Salford Royal NHS Foundation Trust: Smeeta Sinha, Chukwuma Chukwu, Amy Hudson, Diane Campbell, Melanie Kershaw, Stephanie Whittaker, Katarzyna Adeniji; Brighton and Sussex University Hospital's NHS Trust: Ayesha Irtiza-Ali, Farid Ghalli, Heba Nosseir, Allison Leslie, Kate Trivedi; University Hospital of Wales, Cardiff: Donald Fraser, Mohammad Alhadj Ali, Sian Griffin, Farah Latif, Justyna Witczak, Alexa Wonnacott, Lynda Jeffers, Yvette Webley; Edinburgh Royal Infirmary: Paul Phelan, Eve Miller-Hodges, Ailsa Geddes, Margaret Glenwright, Amy Hunter; Gloucestershire Hospitals NHS Foundation Trust: Thomas Pickett, Jim Moriarty, Linda Hill, Amanda Tyler; University Hospitals Coventry and Warwickshire: Waqar Ayub, Gail Evans, Sue Hewins, Davina Hewitt, Kerry Read; Ninewells Hospital: Samira Bell, Leanne Cosgrove, Rachel Craik, Shona Murray; Royal Berkshire Hospital, Reading: Nitin Bhandary, Holly Coles, Rashmi Easow, Maya Joseph, Deepa Thapa; Northern General Hospital, Sheffield: Arif Khwaja, Yvonne Jackson, Angeline Mbuyisa, Rachel Sellars, Sadaf Younis, Kimiko Chapman; Darent Valley Hospital, Dartford: Nihil Chitalia, Cynthia Mohandas, Anca Gherman, Charlotte Kamundi, Olumide Olufuwa, Ryan Coe; Royal London Hospital: Kieran McCafferty, Adedolapo Adeleke, Cara Healy, Damini Jeyarajah, Edward Kinsella-Perks; Ipswich Hospital: Richard Smith, Brian Camilleri, Carol Buckman, Jenny Finch, Vanessa Rivers; University Hospitals Plymouth NHS Trust: Andrew Connor, Sheila Carr, Lisa Shainberg; Cheltenham General Hospital: Thomas Pickett, Linda Hill, Amanda Tyler; St. James's University Hospital, Leeds: Andrew Lewington, Richard Baker, Suzannah Dorey, Kay Tobin, Rosalyn Wheatley; St. George's University Hospitals NHS Foundation Trust: Debasish Banerjee, Richard Hull, Sharirose Abat, Riny Paul; Norfolk and Norwich University Hospitals: Mahzuz Karim, Zay Htet, Rotimi Oluyombo, Saad Tufail, Ravi Varma, Karen Convery, Deirdre Fottrell-Gould, Lisa Hudig, Emily Tropman, Jane Platt; Walsall Healthcare NHS Trust: Thahir Abdul-Samad, Anne Grace, Marie Phipps, Gemma Highway; St Helier Hospital, Carshalton: Rebecca Suckling, Subash Somalanka, Bhrigu Sood, Pauline Swift, Sarah Acheampong, Kwame Ansu, Martia Augustin; Wessex Kidney Centre, Queen Alexandra Hospital, Portsmouth: Anna Sampson, Lynn Vinall, Kim Wren; St Bartholomew's Medical Centre: Shamila Wanninayake, Nicholas Wooding, Heather Edwards, Lydia Owen; Antrim Area Hospital: Stephanie

Bolton, Marion Carson, Michael Matthews; University Hospitals of Leicester: Nigel Brunskill, Jorge Jesus-Silva, Alex Howson, Mary Quashie-Akponeware, April Maria Murillo; North Middlesex University Trust Diabetes Department, North Middlesex University Hospital: Hilary Tindall, Chidambaram Nethaji, Helen Eldon; Glasgow Clinical Research Facility, Queen Elizabeth University Hospital: Rajan Patel, Patrick Mark, Alastair Rankin, Michael Sullivan, Kirsty Forsyth, Rowan McDougall; Great Western Hospital, Swindon: Tanaji Dasgupta, Louisa Davies, Maggie Ryder, Suzannah Pegler; Hathaway Medical Centre, Chippenham: Philip Grimmer, Clare Macdonald, Mary Webster; Newcastle: Timothy Ellam, Edwin Wong, Christine Meshykhi, Andrea Webster, Peter Wilson; Lister Hospital: Enric Vilar, Jocelyn Berdeprado, Eunice Doctolero, Lily Wilkinson; Altnagelvin Hospital, Western Health & Social Care Trust: Frank McCarroll, Hesham Ammar, Ying Kuan, Conor Moran, Girish Shivashankar, Ryan Campbell, Deborah Glowski, Paula McDermott; Oakenhurst Medical Practice, Blackburn: Amar Ali, Zuber Patel, Christine Bond, Gillian Whalley

**China:** National Clinical Research Center of Kidney Diseases, Jinling Hospital, Nanjing University School of Medicine: Haitao Zhang, Peiling Chen, Yu An, Yinghua Chen, Liu Yang, Lihua Zhang, Tingting Kan, Ling Zhu; The Second Affiliated Hospital of Army Medical University, PLA: Jinghong Zhao, Weiping Hou, Jing Wu; Beijing Anzhen Hospital, Capital Medical University: Hong Cheng, Weijing Bian, Zhirui Zhao; Henan Provincial People's Hospital: Fengmin Shao, Huixia Cao, Xiaojing Jiao, Peiyuan Niu; Shanghai Fifth People's Hospital, Fudan University: Jianying Niu, Yu Chen, Lihong Zhang; Huazhong University of Science and Technology Union Shenzhen Hospital: Shenglang Zhu, Haiyan Lin, Shaopeng Yao, Jiehui Chen, Ying Jiang; The second affiliated hospital of Zhejiang University School of Medicine: Ying Hu, Huaying Xiao, Fuye Yang; Shenzhen People's Hospital: Xinzhou Zhang, Baochun Guo, Qiu Jin, Lixia Liu; Xiangya Hospital, Central South University: Xiangcheng Xiao, Yanyun Xie, Ting Meng; Wuhan Fourth Hospital: Chuanwen Xu, Jie Huang, Yanmei Xu; Suzhou Kowloon Hospital: Weixin Kong, Xiaoliang Wang, Qianpan Liu,; Jinzhou Central Hospital: Xueying Wang, Ming Gao; Zhuzhou Central Hospital (Nephrology): Xiumei Hu, Ying Lu; Sichuan Provincial People's Hospital: Li Wang, Kun Peng, Wei Wang; Fuwai Hospital, Chinese Academy of Medical Sciences: Qiuhong Gong, Jianfang Cai, Xiaojue Li, Xuejiao Liu, Haitao Zhang, Shuhan Zhou; Zhuzhou Central Hospital (Endocrinology): Hong Liu, Yao Weng, Shuai Tang, Yao Yao; The Central Hospital of Wuhan: Shi Zhao, Chen Cheng, Wei Wei, Na Li

**Malaysia:** Hospital Kajang: Sadanah Aqashiah Mazlan, Alia Zubaidah Bahtar, Elliyyin Katiman, Noraini Othman; Hospital Tuanku Ja'afar: Lily Mushahar, Nurdiana Mazlan, Nur Sharafina Safiee, Sarasa Ramasamy; Hospital Selayang: Hin Seng Wong, Hajar Ahmad Rosdi, Esther Zhao Zhi Tan, Ju Fan Tay; Hospital Taiping: Kok Seng Teng, Hasnah Yahaya; Hospital Sultanah Aminah: Wen Jiun Liu, Lik Wee Ee, Kenneth Kay Leong Khoo, Yuana Mohd Yusoff; Hospital Tengku Ampuan Afzan: Fariz Safhan Mohamad Nor, Mohd Kamil Ahmad, Mohd Ramli Seman; Hospital Umum Sarawak: Clare Hui Hong Tan, Laura Lui Sian Ngu, Jaime Yoke May Chan, Javelin Peji; Hospital Raja Permaisuri Bainun: Chek Loong Loh, Yee Yan Lee, Sridhar Ramanaidu, Kah Mean Thong, Yik Hong Wong, Suria Junus; Hospital Sultanah Bahiyah: Chen Hua Ching, Mohammad Faisal Asmee, Ku Ruziana Ku Md Razi, Chun Leong Low, Christopher Sze Bing Sim, Zhang Duan Tham, Noor Kamila Abdullah; Hospital Sultan Abdul Halim: Tai Meng Chen, Yong Chieh Chan, Eason Chang, Huan Yean Kang, Kai Quan Lee, Sue Ann Lee, Aik Kheng Lee, Jeevika Vinathan, Chyi Shyang Tan; Universiti Kebangsaan Malaysia Medical Centre: Rizna Abdul Cader, Ruslinda Mustafar, Lydia Kamaruzaman, Rozita Mohd, Rahimah Ismail; Hospital Kulim: Chong Men Leong, Chee Koon Low, Liang Wei Wong, Yik Shen Lim, Norlezah Adnan, Sabariah Ibrahim; Hospital Kuala Lumpur: Mohamad Zaimi Abdul Wahab, Sunita Bavanandan, Yik Shen Lim, Zhang Duan Tham, Wan Hazlina Wan Mohamad, Siti Munirah Jaafar, Nur Ashykeen Mohd Fauzi, Aziee Sudin; University Malaya Medical Centre: Soo Kun Lim, Chye Chung Gan, Albert Hing, Wan Ahmad Faizal Alaidin Razali; Hospital Pulau Pinang: Yew Fong Liew, Chelsia Bao Tyng Chan, Mei Chih Cheng, Yu Chen Ong, Loke Meng Ong, Farah Amalina Mohamed Affandi; Hospital Melaka: Korina Rahmat, Ban Chai Peng, Masayu Amat; Hospital Pakar Sultanah Fatimah: Nuzaimin Hadafi Ahmad, Doo Yee Mah, Yi Loon Tye, Zaid Azhari, Siti Nabilah Mohamad Zaini, Mohd Aidil Musa, Nur Nadzifah Hanim Zainal Abidin, Zher Lin Go; Hospital Ampang: Norazinizah Ahmad Miswan, Rafizanur Ramli, Nor Aziah Ahmad; Hospital Serdang: Bak Leong Goh, Nurul Izah Ahmad, Fairol Huda Ibrahim, Tze Jian Ng, Malini Shanmuganathan, Li Lian

Tay; Hospital Sultanah Nur Zahirah: Zaiha Harun, Salmi Ramli, Nurul 'Ain Yusof, Rossenizal Abd Rahman; Pusat Perubatan UiTM: Muhammad Iqbal Abdul Hafidz, Nur Hidayati Mohd Sharif, Irda Yasmoon Awang

**Japan:** Chubu Rosai Hospital: Eitaro Nakashima, Rui Imamine, Makiko Minatoguchi, Yukari Miura, Miduki Nakaoka, Yoshiki Suzuki, Hitomi Yoshikawa; Shin Clinic: Koki Shin, Kanae Fujita, Misuzu Iwasa, Haruka Sasajima, Airi Sato; Kansai Electric Power Hospital: Yoshiyuki Hamamoto, Yuki Fujita, Takuya Haraguchi, Takanori Hyo, Kiyohiro Izumi, Toshiyuki Komiya, Sodai Kubota, Takeshi Kurose, Hitoshi Kuwata, Susumu Nakatani, Kaori Oishi, Saki Okamoto, Kaori Okamura, Jun Takeoka, Nagaaki Tanaka, Katsuya Tanigaki, Naohiro Toda, Koin Watanabe, Hiromi Komori, Rika Kumuji, Asako Takesada, Aya Tanaka; Nagoya University Hospital: Shoichi Maruyama, Tomonori Hasegawa, Akiko Ishiguro, Takuji Ishimoto, Kazuhiro Ito, Yutaka Kamimura, Noritoshi Kato, Sawako Kato, Hiroshi Kojima, Tomoki Kosugi, Kayaho Maeda, Masasi Mizuno, Shoji Saito, Hitomi Sato, Yuka Sato, Yasuhiro Suzuki, Akihito Tanaka, Yoshinari Yasuda, Fujiko Hasegawa, Maiko Hayashi, Shizuka Higashi, Kaho Shimamura, Momoko Sumi, Kazuki Tajima, Chimaki Unekawa, Kana Wakayama, Yukiko Wakita; Ota diabetes clinic: Takatoshi Otani, Ayako Imai, Sayaka Kawashima, Eri Kogure, Tomoe Sato, Misato Takezawa, Shinya Yoshida; Fukui Prefectural Hospital: Hideo Araki, Yuko Katsuda, Masahiro Konishi, Takahiro Matsunaga, Masashi Oe, Kunihiro Ogane, Masato Sakai, Tomoko Takahashi, Takahiro Yamano, Takuya Yokoyama, Hitomi Ito, Masayo Katayama, Emi Kuroda; Medical Corporation Seijinkai Ikeda Hospital: Toru Ikeda, Takuma Kojo, Etsuo Yoshidome, Rieko Mizumachi, Akane Yamamoto, Narihisa Yamasaki, Yoshihiko Yamasaki; Okayama University Hospital: Jun Wada, Jun Eguchi, Chigusa Higuchi, Akihiro Katayama, Masaru Kinomura, Masashi Kitagawa, Shinji Kitamura, Satoshi Miyamoto, Hiroshi Morinaga, Atsuko Nakatsuka, Ichiro Nojima, Kenichi Shikata, Hitoshi Sugiyama, Katsuyuki Tanabe, Kenji Tsuji, Haruhito Uchida, Mayu Watanabe, Chie Hashimoto, Takahiro Kato, Sayaka Yamamoto; Tokai University Hospital: Takehiko Wada, Masafumi Fukagawa, Naoto Hamano, Masahiro Koizumi, Hirotaka Komaba, Yosuke Nakagawa, Michiyo Iwamoto; Fukuoka University Hospital: Kosuke Masutani, Akane Katanosaka, Mayu Kiyota, Hikari Uchi, Yuka Ueda, Sonoka Yamamoto; Kawasaki Medical School Hospital: Hajime Nagasu, Seiji Itano, Tsukasa Iwakura, Hiroyuki Kadoya, Eiichiro Kanda, Naoki Kashihara, Kengo Kidokoro, Megumi Kondo, Tamaki Sasaki, Minoru Satoh, Atsuyuki Tokuyama, Reina Umeno, Yoshihisa Wada, Toshiya Yamamoto, Yu Yamanouchi, Masumi Abe, Yoko Inukai; Kobe University Hospital: Wataru Ogawa, Shunichiro Asahara, Hideki Fujii, Shunsuke Goto, Yushi Hirota, Tetsuya Hosooka, Keiji Kono, Shinichi Nishi, Yuko Okada, Kazuhiko Sakaguchi, Kenji Sugawara, Michiko Takahashi, Tomoko Takai, Yoshikazu Tamori, Kentaro Watanabe, Miyu Kitajima, Misaki Nishi, Junko Wada; Aichi Medical University Hospital: Yasuhiko Ito, Hideki Kamiya, Akimasa Asai, Nao Asai, Saeko Asano, Shogo Banno, Yohei Ejima, Hanako Hase, Tomohide Hayami, Tatsuhito Himeno, Takahiro Ishikawa, Mayumi Ito, Shiho Iwagaitsu, Rina Kasagi, Yoshiro Kato, Makoto Kato, Koichi Kato, Takayuki Katsuno, Miyuka Kawai, Hiroshi Kinashi, Masaki Kondo, Masako Koshino, Naoya Matsuoka, Yoshiaki Morishita, Mikio Motegi, Jiro Nakamura, Hiromi Shimoda, Hirokazu Sugiyama, Shin Tsunekawa, Makoto Yamaguchi, Kazuyo Takahashi; Juntendo University Hospital: Hirotaka Watada, Takashi Funayama, Yasuhiko Furukawa, Tomohito Gohda, Hiromasa Goto, Hideyoshi Kaga, Yasuhiko Kanaguchi, Akio Kanazawa, Kayo Kaneko, Toshiki Kano, Masao Kihara, Shogo Kimura, Takashi Kobayashi, Masayuki Maiguma, Yuko Makita, Satoshi Mano, Tomoya Mita, Takeshi Miyatsuka, Maki Murakoshi, Masahiro Muto, Masami Nakata, Junichiro Nakata, Yuya Nishida, Nao Nohara, Takeshi Ogihara, Daisuke Sato, Junko Sato, Hiroaki Sato, Yusuke Suzuki, Ruka Suzuki, Hitoshi Suzuki, Miyuki Takagi, Yoshifumi Tamura, Toyoyoshi Uchida, Seiji Ueda, Miki Asawa, Minako Miyaji, Eri Nagashima, Yoshie Shibata, Eri Yanagisawa; The University of Tokyo School of Medicine/Toranomon Hospital: Takashi Kadowaki, Toshimasa Yamauchi, Masaomi Nangaku, Yosuke Hirakawa, Hiroshi Nishi, Nobuhiro Shojima, Satoko Horikawa, Yukiko Nakayama, Naoko Yamada, Yuki Omori; Maebashi Hirosegawa Clinic: Shintaro Yano, Miyabi Ioka, Nahoko Kuwabara, Remi Nagano, Megumi Nozawa, Yumi Osawa; Shiga University of Medical Science Hospital: Hiroshi Maegawa, Shinji Kume, Shinichi Araki, Itsuko Miyazawa, Katsutaro Morino, Ikuko Kawai, Masumi Sobata, Motoko Takaoka; Koukan Clinic: Yasushi Iwaita, Takashi Udagawa, Ami Inamori, Aya Kawase, Aya Yamanaka; University of Tsukuba Hospital: Hitoshi Shimano, Akiko Fujita, Hitoshi Iwasaki, Hirayasu Kai, Yoshinori Osaki, Chie Saito, Motohiro Sekiya, Ryoya Tsunoda, Kunihiro Yamagata, Rikako

Nakamura, Aiko Yamada; Center Hospital of the National Center for Global Health and Medicine: Mitsuru Ohsugi, Motoharu Awazawa, Ryotaro Bouchi, Shota Hashimoto, Makiko Hashimoto, Tomoko Hisatake, Noriko Ihana, Koko Ishizuka, Kazuo Izumi, Hiroshi Kajio, Michi Kobayashi, Noriko Kodani, Koji Maruyama, Michihiro Matsumoto, Maya Matsushita, Tomoka Nakamura, Takehiro Sugiyama, Akiyo Tanabe, Aiko Terakawa, Kojiro Ueki, Yuko Orimo, Takako Ozawa, Eriko Takahira; AMC Nishi-Umeda Clinic: Yoshimitsu Yamasaki, Masakazu Haneda, Tadahiro Tomita, Saori Akimoto, Akihiro Fujimoto, Kenji Ishihara, Chiho Murakami, Akiyo Nishiyama, Yukiko Toyonaga, Kana Uozumi, Yukihiro Yamaji; Jyoumou Ohashi Clinic: Tetsuya Shigehara, Jun Okajyo, Yukihiro Shimizu; Iwasaki internal medicine clinic: Shingo Iwasaki, Yuki Fukao, Megumi Furusho, Shintaro Nunokawa; Tohoku University Hospital: Hideki Katagiri, Tomohito Izumi, Keizo Kaneko, Shinjiro Kodama, Mariko Miyazaki, Yuichiro Munakata, Tasuku Nagasawa, Yuji Oe, Hiroto Sugawara, Kei Takahashi, Kazushige Hirata, Keiko Inomata, Shoko Otomo, Taeko Uchida, Chigusa Yamashita; Tokyo-eki Center-building Clinic: Arihiro Kiyosue, Ryota Tamura

**Canada:** CRIUCPQ: Francois Dube, Marilene Bolduc, Marie-Christine Talbot; University Health Network-Toronto General Hospital: David Cherney, Leslie Cham, Vesta Lai, Josephine Tse; Clinical Research Solutions Inc.: Shivinder Jolly, Tabbatha Duck; Interior Health Kelowna General Hospital: Scott Lyle, Rachel Epp, Camille Galloway, Susan Haskett, Elizabeta Matvienko, Liam Paulsen, Zachary Walbaum; London Health Sciences Centre: Louise Moist, Kerri Gallo, Zabrina Lozon, Tina Ramsey, Brittany Whitmore; St Paul's Hospital: Adeera Levin, Bader Al-Zeer, Paula Macleod, Aoife O'Sullivan, Zainab Sheriff, Sam Tholl; Cambridge Cardiac Care Centre: Amritanshu Pandey, Samantha Armstrong, Bethelihem Gebeyehu, Patrick Toth; LMC Clinical Research Inc. (Thornhill): Ronald Goldenberg, Mahsa Jahangiriesmaili, Shariff Sanguila, Neethi Suresh, Tanvi Talsania; Vancouver General Hospital: Nadia Zalunardo, Bader Al-Zeer, Paula Macleod, Aoife O'Sullivan, Zainab Sheriff; CHU de Quebec-Universite Laval: Mohsen Agharazii, Marie-Pier Roussel, Annie Saillant, France Samson; LMC Clinical Research Inc. Brampton: Harpreet Bajaj, Miken Bhavsar, Parul Dhall, Gagandeep Dhillon, Bhupinder Grewal, Taniya Nimbkar, Radica Richards, Julia Lee; CIUSSS Nord de l'ile de Montreal: Francois Madore, Guylaine Marcotte; LMC Clinical Research Inc. (Bayview): Oren Steen, Mathura Bullen, Shayani Raguwaran, Andre Valleteau; CIUSSS de l'Estrie-CHUS, Hopital Fleurimont: Marie-France Langlois, Christine Brown; Lakeridge Health: Andrew Steele, Melissa Garrity, Taneera Ghate, Holly Robinson, Michael Tolibas; LMC Clinical Research Inc. (Ottawa): Chetna Tailor, Lauren Elliott, Christine McClary-Wright; Fadia El Boreky Medicine Professional: Fadia Boreky, Sameh Fikry, Ayesha Ali, Chintankumar Barot, Wagdy Basily, Bethelihem Gebeyehu, Thisun Saram, Vinay Varad, Karimula Mogal; LMC Clinical Research Inc (Etobicoke): Hasnain Khandwala, Alex Aguilera, Patricia Alvarez, Balwinder Gill, Nazihah Huda, Aamir Navivala, Daniel Pinto, Hitu Sharma; Kidney Care Centre-Fraser Health: Micheli Bevilacqua, Elaine Fung, Geraldine Hernandez, Puneet Mann, Jaskiran Saini, Natasha Curtis; Institut de recherches cliniques de Montreal: Remi Rabasa-Lhoret, Danijela Bovan, Marie Devaux

**Italy:** Policlinico San Martino, Genova: Roberto Pontremoli, Cecilia Barnini, Giovanna Leoncini, Luca Manco, Giulia Nobili; Ospedale Casa Sollievo della Sofferenza, San Giovanni Rotondo: Matteo Piemontese, Filippo Aucella, Rachele Grifa, Francesco Totaro; Policlinico S. Orsola-Malpighi, Bologna: Gaetano La Manna, Irene Capelli, Giuseppe Cianciolo, Sarah Lerario, Fulvia Zappulo; Ospedale S. Giovanni di Dio, Firenze: Alberto Rosati, Filippo Fani, Giuseppe Spatoliatore, Ester Baldini, Francesca Bianchini; AOU Policlinico, Bari: Loreto Gesualdo, Francesco Pesce, Maria Russo, Maria Zippo, Cesira Cafiero, Maria Ficarella, Marica Romano; Ospedale Martini, Torino: Daria Motta, Simona Bianco, Donatella Bilucaglia; Ospedale Maggiore Policlinico, Milano: Piergiorgio Messa, Laura Pavone, Federica Tripodi, Simone Vettoretti, Giuseppe Castellano, Emilietta Brigati; AOU Padova: Paola Fioretto, Gianni Carraro, Filippo Farnia, Anna Postal; Ospedale Sacro Cuore di Gesù, Gallipoli: Alessandro D'Amelio, Antonio Cardone, Giovanni Piccinni, Annalisa Aloisi; ASST Spedali Civili, Brescia: Francesco Scolari, Federico Alberici, Alice Guerini, Chiara Saccà, Chiara Salviani, Roberta Zani; AOU L. Vanvitelli, Napoli: Luca De Nicola, Carlo Garofalo, Maria Elena Liberti, Roberto Minutolo, Luigi Pennino, Lucio Polese; AOU Sant' Andrea, Roma: Paolo Mené, Simona Barberi, Clorinda Falcone; Ospedale Ignazio Veris delli Ponti, Scorrano: Francesco Russo, Maurizio Caroppo; Ospedale di Circolo, Desio: Gennaro Santorelli, Rodolfo Rivera; AOU Policlinico G.

Martino, Messina: Domenico Santoro, Alfio Giuffrida, Fortunata Zirino, Roberto Gallo ; Ospedale Civile SS. Antonio e Biagio, Alessandria: Cristina Calvi, Luca Estienne; AOUI, Verona: Giovanni Gambaro, Concetta Gangemi, Vittorio Ortalda, Giuseppina Pessolano; Fondazione Policlinico Universitario Agostino Gemelli, Roma: Giuseppe Grandaliano, Rocco Baccaro, Pietro Ferraro, Roberto Mangiacapra; IRCCS Ospedale San Raffaele, Milano: Marco Melandri, Nadia Foligno, Rita Quartagno, Giuseppe Vezzoli, Elena Brioni, Paola Maiucchi, Tunesi Francesca

# Supplementary Methods

## EMPA-KIDNEY definitions of end-stage kidney disease and approaches to verification of deaths using medical notes (clinical adjudication)

## (A) END-STAGE KIDNEY DISEASE (ESKD)

### *Definition*

According to the main trial Protocol, the definition of ESKD includes:

i. Initiation of maintenance dialysis;
ii. Receipt of a kidney transplant.

### Initiation of maintenance dialysis

At each visit, information was sought about initiation of dialysis. When reporting dialysis, investigators are asked to provide the date dialysis started and:

i. Confirm whether dialysis is ongoing or was temporary (and if only temporary, the reason for stopping). In general, ongoing dialysis is considered as maintenance dialysis if it is required for ≥90 days. Note that death within 90 days of starting dialysis is a special situation.[1]
ii. Confirm the type of dialysis (peritoneal dialysis or hemodialysis).

### Receipt of a kidney transplant

At each visit, information was sought about kidney transplantation. When reporting receipt of a kidney transplant, investigators are asked to confirm the date of the procedure.

## (B) DEATHS

### *Approach to adjudication*

All reported deaths were reviewed and medical notes sought from Local Clinical Centers (LCCs). Blinded clinicians based at, or overseen by, the Central Coordinating Office in Oxford provide the final adjudication. These clinicians may include, but will not necessarily be, nephrologists or cardiologists. They work under the guidance of senior clinical trialists with extensive experience of adjudicating many thousands of such events (including ESKD, cardiovascular events, and death) in previous large-scale trials in similar populations.

Adjudication procedures in the post-trial follow-up period were identical to those from the active trial period. In making their final decisions, clinicians review the additional information collected from the LCCs as well as other potentially relevant information recorded during study Follow-up visits. SOP 9b: Adjudication Procedures details a quality control process where an initial random sample of first events assessed by each adjudicator are to be reviewed.

i. All reported deaths were required to be adjudicated to confirm:
    a. The date of death
    b. The cause of death

ii. Deaths are to be coded using MedDRA Preferred Terms. They are categorized to facilitate the protocol-specified analyses as follows:

---

[1] During adjudication of a death, a central clinician adjudicator may note the start of dialysis therapy. The adjudicator is required to provide an opinion on whether the dialysis would have been likely to be required long-term (i.e. renal recovery to be independent of dialysis is unlikely) or only temporarily (i.e. dialysis was started for acute kidney injury and recovery would have been expected had the participant not died within 90 days of starting dialysis).

| | | |
|---|---|---|
| **Cardiovascular death** | | |
| Cardiac | Coronary heart disease | Myocardial infarction<br><br>Other coronary heart disease |
| | Other cardiac disease | Specific cardiac causes, including non-ischemic heart failure<br><br>Sudden cardiac death |
| Stroke | Hemorrhagic<br><br>Ischemic<br><br>Undetermined | <br><br>Ischemic stroke (+/- hemorrhagic transformation) |
| Other cardiovascular | Specific other causes (not listed above) | (e.g. pulmonary embolism, ruptured aortic aneurysm) |
| Presumed cardiovascular | Unexplained death | |
| **Non-cardiovascular death** | | |
| Medical | Renal | Death due to chronic kidney disease stage G5 (kidney failure) |
| | Infection (incl. Covid-19) | |
| | Cancer | |
| | Other medical | |
| Non-medical | External | Trauma |

## Cardiovascular death definitions

Cardiovascular death includes deaths due to any of the following categories: cardiac, stroke, other cardiovascular and presumed cardiovascular (see table above).

**Myocardial infarction death**
Death due to acute myocardial infarction (MI) refers to a death within 30 days after a MI related to consequences seen immediately after the MI, such as progressive congestive heart failure, inadequate cardiac output, or recalcitrant arrhythmia. This includes:

i. Deaths due to either acute MI; and
ii. Deaths due to surgical and non-surgical investigations and procedures to treat acute MI or a complication thereof.

Deaths occurring within 30 days after a MI should be attributed to MI unless there was a clear alternative cause.

***Supporting evidence***
To confirm death from MI there should be evidence of myocardial necrosis plus at least one other piece of supporting information, and no other likely diagnosis:

**i. Evidence of myocardial necrosis from:**
   a. Raised cardiac biomarker results compatible with acute myocardial necrosis (after taking into consideration the potential effects of chronic kidney disease [CKD] on such biomarkers); or
   b. Autopsy with MI or coronary thrombus of an age consistent with the clinical presentation.

**ii. Other supporting information:**
   a. Relevant presentation:
      1. Symptoms of ischemia; or
      2. Death
   b. ECG evidence of:
      1. New ischemia; or
      2. Development of pathological Q waves
   c. Cardiac imaging demonstrating new myocardial defect or evidence of acute coronary occlusion.

**Other coronary heart disease death**
Death due to other coronary disease includes cardiac causes of death that are believed related to coronary atherosclerosis. This includes:

i. Deaths that are related to angina (e.g. death following admission with unstable angina), chronic ischemia (including ischemic cardiomyopathy), and late complications of MI (after 30 days);
ii. Deaths due to surgical and non-surgical investigations and procedures for coronary artery disease (other than as treatments for acute MI); and
iii. Deaths which are believed to be due to coronary atherosclerosis, with autopsy findings of coronary artery disease but without a lesion of an age corresponding to the clinical presentation.

In all cases, deaths due to MI as defined above should be excluded.

**Other cardiac disease death (including sudden cardiac death)**
Death due to other cardiac disease includes:

i. Deaths that are likely to be due to specific cardiac disorders (e.g. valvular heart disease, non-ischemic cardiomyopathy, primary arrhythmia); and
ii. Deaths due to surgical and non-surgical investigations and procedures for other cardiac disease (other than for coronary artery disease);
iii. Deaths that are sudden and believed likely to be due to cardiac disease, but no definite cardiac cause described above has been identified are sudden cardiac deaths (MedDRA Preferred Term: “Sudden

cardiac death" [10049418]). These include witnessed sudden deaths with or without new or worsening of cardiac symptoms, and unwitnessed deaths in a participant who was seen to be alive and clinically well within 72 hours of being found dead without any evidence of another cardiovascular or a non-cardiovascular cause.

Other cardiac death excludes deaths thought to be due to coronary heart disease (including MI). In some cases, there may be evidence of both coronary heart disease and other cardiac disease (e.g. coronary atherosclerosis in a patient with aortic stenosis). In such cases, the available evidence (e.g. clinical presentation, eye-witness reports, clinical investigations, autopsy findings) should be used to determine the pathology that most likely led to death.

**Stroke death**

Stroke is defined as an acute episode of focal or global neurological dysfunction caused by brain, spinal cord, or retinal vascular injury as a result of hemorrhage or infarction. Death due to stroke refers to a death which results from:

i. An inexorable decline in the condition of the patient following stroke (typically, but not always within 30 days of the initial event); or
ii. A complication of the stroke (e.g. infection, complication of intervention or procedure); or
iii. Withdrawal of other therapies because of concerns relating to the poor prognosis associated with the stroke (e.g. withdrawal of dialysis).

Wherever possible, stroke subtype (e.g. hemorrhagic, ischemic or undetermined) should be differentiated.

***Hemorrhagic stroke***

Hemorrhagic stroke is defined as a stroke caused by a non-traumatic intraparenchymal, intraventricular, or subarachnoid hemorrhage.

***Ischemic stroke***

For the purposes of analysis, ischemic stroke will include all of the following:

i. Ischemic stroke, which is defined as a stroke caused by an infarction of central nervous system tissue; or
ii. Ischemic stroke with hemorrhagic transformation, which is defined as ischemic stroke with evidence of subsequent hemorrhage into an area of previous infarction.

***Undetermined stroke***

Undetermined stroke is defined as a stroke of unknown/unconfirmed pathological type, i.e. a stroke for which it is unclear whether there is an ischemic or hemorrhagic cause because imaging was not performed or the result is not available.

***Exclusions***

The following conditions are not to be included in the definition of stroke:

i. Severe prolonged hypoglycemia resulting in neurological deficits;
ii. Trauma;
iii. Any subdural or extradural hematoma;
iv. Findings on CT scans (done for any reason) that do not correlate with a clinical episode;
v. Transient ischemic attacks and related syndromes (with symptoms lasting <24 hours and not leading to death, unless there is clear evidence of new infarction on cranial imaging); and
vi. Non-cardiovascular pathologies (e.g. tumor, degenerative disorders).

**Other cardiovascular death**

Other cardiovascular death includes:

i. Deaths due to other cardiovascular causes (e.g. pulmonary embolism, primary pulmonary hypertension, ruptured aortic aneurysm, limb ischemia); and

ii. Deaths due to surgical and non-surgical investigations and procedures for other cardiovascular disease (including peripheral arterial revascularization procedures such as peripheral bypass grafting, endarterectomy, arterectomy, embolectomy, and angioplasty).

**Unexplained death**

i. Deaths for which no cause can be determined in a CKD population are often from cardiovascular causes, and are to be considered presumed cardiovascular deaths.[2]
ii. Deaths which are unexplained are those with no evidence of an alternative cardiovascular or non-cardiovascular cause of death (excluding sudden cardiac death – see Section 3.2.3). This would include deaths for which the cause of death is medically unclear (despite adequate supporting documentation) as well as those for which there is inadequate documentation (despite best efforts). [For example, a death for which there is no information beyond "found dead at home" or "patient died", despite efforts to obtain further details (e.g. medical records, witness report) may be adjudicated as unexplained death.]

## Non-cardiovascular death definitions

Non-cardiovascular death includes deaths due to any of the following categories: renal, infection, cancer, other medical and non-medical. Discussion with a Principal Investigator is encouraged for difficult cases. This may be particularly important if there is uncertainty as to whether a cardiovascular or a non-cardiovascular disease was the predominant cause of death.

**Renal death**

Evidence of renal death requires:

i. Evidence of CKD stage 5 (i.e. eGFR <15 mL/min/1.73m$^2$ [and usually <10 mL/min/1.73m$^2$] or ESKD); AND
ii. Evidence of:
    a. Conservative management of ESKD: the patient or their representatives had decided that, despite a clinical need, kidney failure replacement therapy [KFRT] was not to be provided. This includes progression to ESKD before KFRT can be provided; OR
    b. Withdrawal from KFRT: the patient or their representative has chosen to discontinue KFRT (e.g. personal choice, intolerant of dialysis therapy); AND

    No evidence that other major pathology led: (a) to death, or (b) to the decision to withdraw from KFRT (e.g. cancer or stroke), or (c) made dialysis infeasible. Such deaths should be ascribed to the underlying condition.

**Infection death**

i. Infection should be coded as the cause of death if:
    - death results directly from the infection;
    - death results from a complication of the infection (e.g. acute kidney injury).
ii. Infection should <u>not</u> be coded as the cause of death if the criteria for renal death, cancer death or any type of cardiovascular death are met (e.g. death resulting from pneumonia occurring 2 weeks after a stroke would be coded as a stroke death).

**Cancer death**

The underlying cause of cancer should be coded as the cause of death if death results:

- Directly from the cancer; or
- From a complication of the cancer or its treatment (e.g. infection / surgery / chemotherapy / radiotherapy); or
- From withdrawal of other therapies (including dialysis) because of concerns relating to the poor prognosis associated with the cancer.

[2] As recommended by Standardized Data Collection for Cardiovascular Trials Initiative (SCTI) Cardiovascular and Stroke Endpoint Definitions for Clinical Trials.

Cancer causes of death are to be categorized by site (e.g. primary organ of origin). Cancer deaths may arise from cancers that were present prior to randomization or which developed subsequently. These two scenarios are to be distinguished in the adjudication process, but if this distinction is not possible (e.g. due to lack of sufficiently detailed documentation) then the cancer will be assumed to have developed after randomization.

**Other specific medical causes of death**

i. The available evidence should be used to determine the most likely cause of death.
ii. Other medical causes of death should not be coded as the cause of death if the criteria for renal death, infection death, cancer death or any type of cardiovascular death are met.
iii. Acute kidney injury, ketoacidosis and liver causes of death should be adjudicated in line with the specific safety outcome definitions (see Supplementary appendix to N Engl J Med 2023;388: 117-127)
iv. Deaths due to surgical and non-surgical investigations and procedures for non-vascular diseases (e.g. bowel resection for Crohn's disease) should be attributed to the underlying disease for which the investigation or procedure was carried out.

**Non-medical death**

Non-medical death is defined as any death that is thought to be due to a non-medical ("external") cause. Not only are such deaths uncommon in most clinical trials, but they are generally unlikely to be affected by study treatments. Examples include suicide, homicide, road traffic accident, house fire, electrocution, war and natural disaster.

## Supplementary Statistical Methods
## (including handling and analysis of estimated GFR measurements)

An estimated GFR was sought in each 6 monthly post-trial follow-up period. Local clinic center staff were trained to record the latest outpatient result in the period. Local outpatient measurements of creatinine instead of measurements during hospitalizations were requested as these are most reflective of the participant's usual kidney function. Local creatinine results could be collected from outpatient clinic visits, general practitioner/primary care practitioner visits, or hospitalization visits (as a last resort).

### Time-to-first event analyses

All statistical analysis methods were pre-specified in Data Analysis Plans. Estimated GFR was calculated from locally measured creatinine using the race-adjusted 2009 CKD-EPI formula. If multiple estimated GFR measurements were available in any one follow-up period, then the estimated GFR closest to the middle of the window was automatically selected. This ensures only a single estimated GFR measurement was selected in each follow-up window, minimizing any bias that could be introduced by any difference in frequency of creatinine measurement between groups. If there are two estimated GFRs recorded in the last follow-up period, the last of these estimated GFRs was included in analyses irrespective of whether or not it is the estimated GFR closest to the middle of the follow-up window. This could result in two estimated GFRs in the last follow-up period.

For the sustained ≥40% estimated GFR decline from randomization, and for the sustained estimated GFR below 10 mL/min/1.73m$^2$ components of the kidney disease progression composite outcome, confirmation of "sustained" required values on two consecutive estimated GFR measurements at least 30 days apart, or was assumed if it was the last estimated GFR value before death, withdrawal of consent, or the end of a participant's follow-up. There was no imputation for missing creatinine values in time-to-first event analyses.

In the primary analyses of the entire follow-up period (active and post-trial periods combined), the analyses from the reported results for the active trial's primary outcome using centrally measured estimated GFR were carried over. A sensitivity analysis using only local estimated GFR measurements collected throughout the entire trial follow-up period was pre-specified and conducted. This analysis did not differentiate the active versus post-trial periods (i.e. in this sensitivity analysis, estimated GFR values at the last scheduled active trial visit were not designated as the "last scheduled visit" for the purposes of "sustained" definitions unless the participant did not enter the post-trial follow-up period in which case it was the participant's actual last visit). A second sensitivity analysis limited all data to those centres participating in post-trial follow-up.

### Estimates of absolute benefits

Absolute benefits per 1000 participants allocated empagliflozin at given time points were calculated from differences in Kaplan-Meier curves between allocated groups at those time points.

Mathematically, if there had been no off-treatment effect of empagliflozin post-trial, absolute benefits would be observed to diminish from the end of the active trial period. This is expected as: let $S_i(t)$ denote the survival function in each allocated group at time t. The difference in the survival curves between the allocated groups at the end of the active period (2.5 years) is denoted by $d = S_1(2.5) - S_2(2.5)$. Due to the beneficial effects of active drug, there will be a separation of the curves at the end of the active trial, with a larger proportion of patients still event free in the empagliflozin group. In the post-trial period, we would expect the proportion of patients still event free in each group to reduce by a factor of $(100-x_i)/100$ each year, where $x_i$ is the hazard rate in arm i. Therefore, n years after the end of the active trial period, the survival function in arm i will be $S_i(2.5)*((100-x_i)/100)^n$. When the post-trial hazard ratio is 1.0, the hazard rates in each arm are identical (i.e. $x_i$=x). In this scenario, the difference

in the survival curves n years after the end of the active trial period would be $S_1(2.5)*((100-x)/100)^n$ – $S_2(2.5)*((100-x)/100)^n = (S_1(2.5)- S_2(2.5))*((100-x)/100)^n = d*((100-x)/100)^n$. Hence, we would expect the absolute benefit observed at the end of the trial period to have diminished by a factor of (100-x)/100 for each year of post-trial follow-up in the absence of any additional off-treatment effect of study drug.

## Mixed model repeated measures (MMRM) approach

Linear MMRM analyses were used to estimate mean estimated GFR by treatment allocation at each scheduled follow-up visit (and these values are shown in Figure S6). These models were adjusted for baseline estimated GFR (as a continuous variable), age, sex, prior diabetes, urinary albumin-to-creatinine ratio, and region (all in the same categories used in the minimization process), treatment allocation, follow-up time point and the interaction between baseline estimated GFR and follow-up time point. A further interaction term between treatment allocation and follow-up time point was then included in order to enable separate estimation of mean estimated GFR at each follow-up time point for each treatment arm, conditional on the other factors in the model. These models assume that any missing estimated GFR values can be predicted by the non-missing estimated GFR data for other individuals together with the other covariates in the model (i.e. that they are ‘missing at random’).

## Supplementary Figures

**Figure S1.** CONSORT participant flowchart for active and post−trial follow-up periods (PTFU)

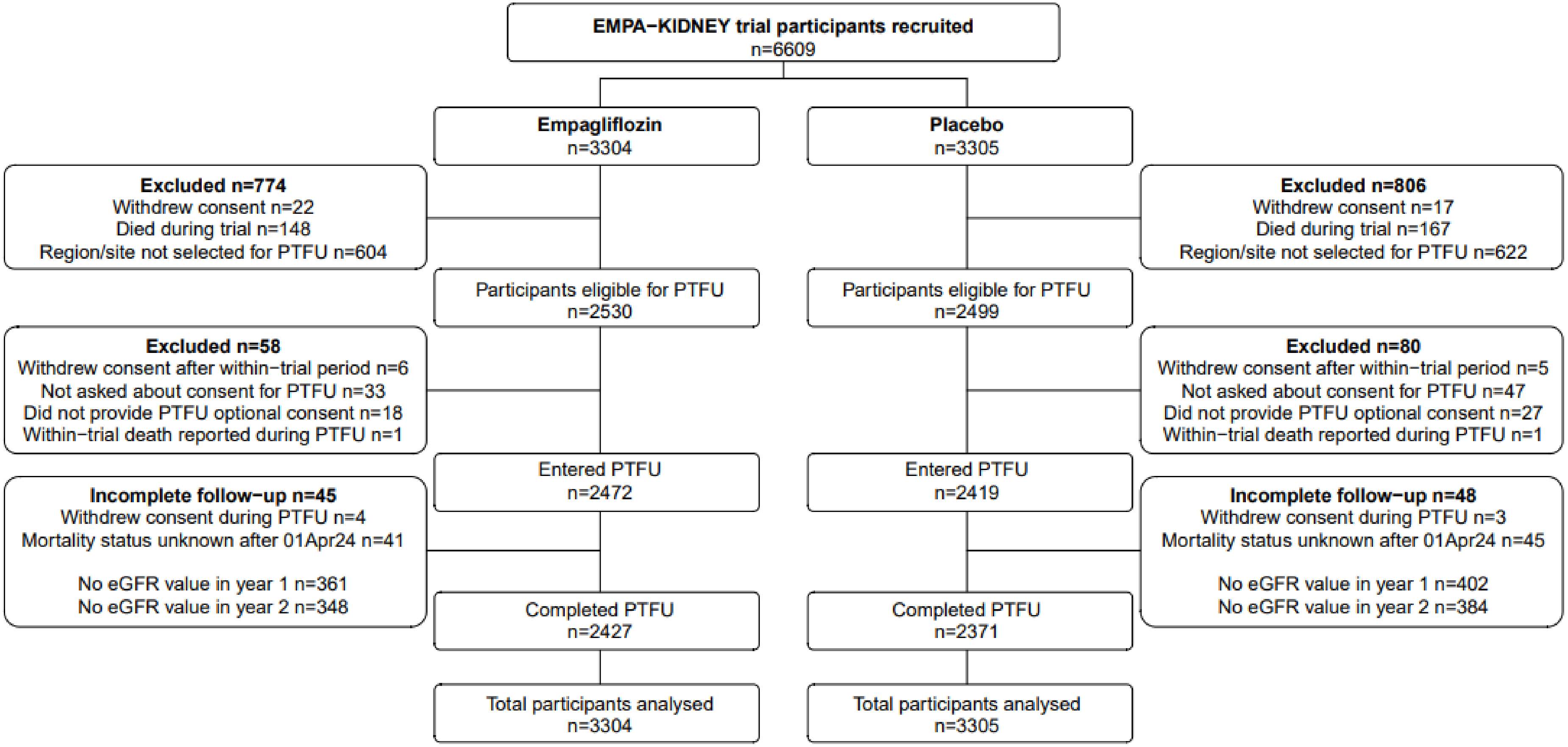


Proportions of participants entering PTFU were: Empagliflozin group: 2472/3304 (74.8%) and placebo group 2419/3305 (73.2%).

**Figure S2:** Effect of allocation to empagliflozin on progression of kidney disease or death from cardiovascular causes, restricted to sites that contributed to post-trial follow-up (sensitivity analysis)

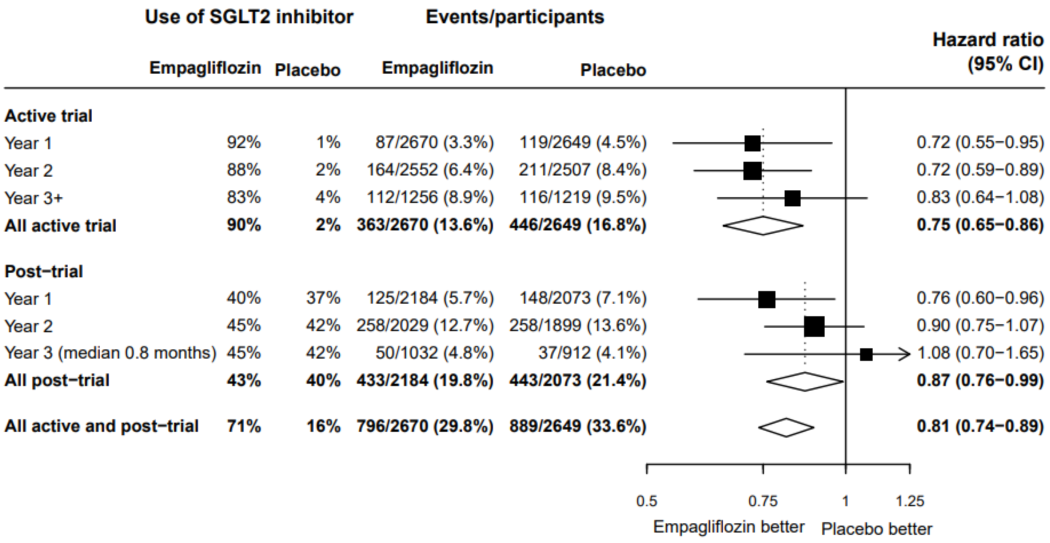


Presented analyses carry over the main results of the trial’s primary outcome (i.e. any estimated glomerular filtration rate [eGFR] based outcome recorded at the active trial period’s Final Follow−up Visit without a confirmatory result are still accepted as “sustained” despite the availability of local eGFR measurements in the post−trial period). Use of SGLT2 inhibitor defined in Table 2 and average use calculated as per Figure 1B with weights proportional to the total person years at risk in each year. Denominators are the number of participants still at risk of a first primary outcome at the start of the risk period.

**Figure S3:** Effect of allocation to empagliflozin on secondary outcomes over the entirety of follow-up

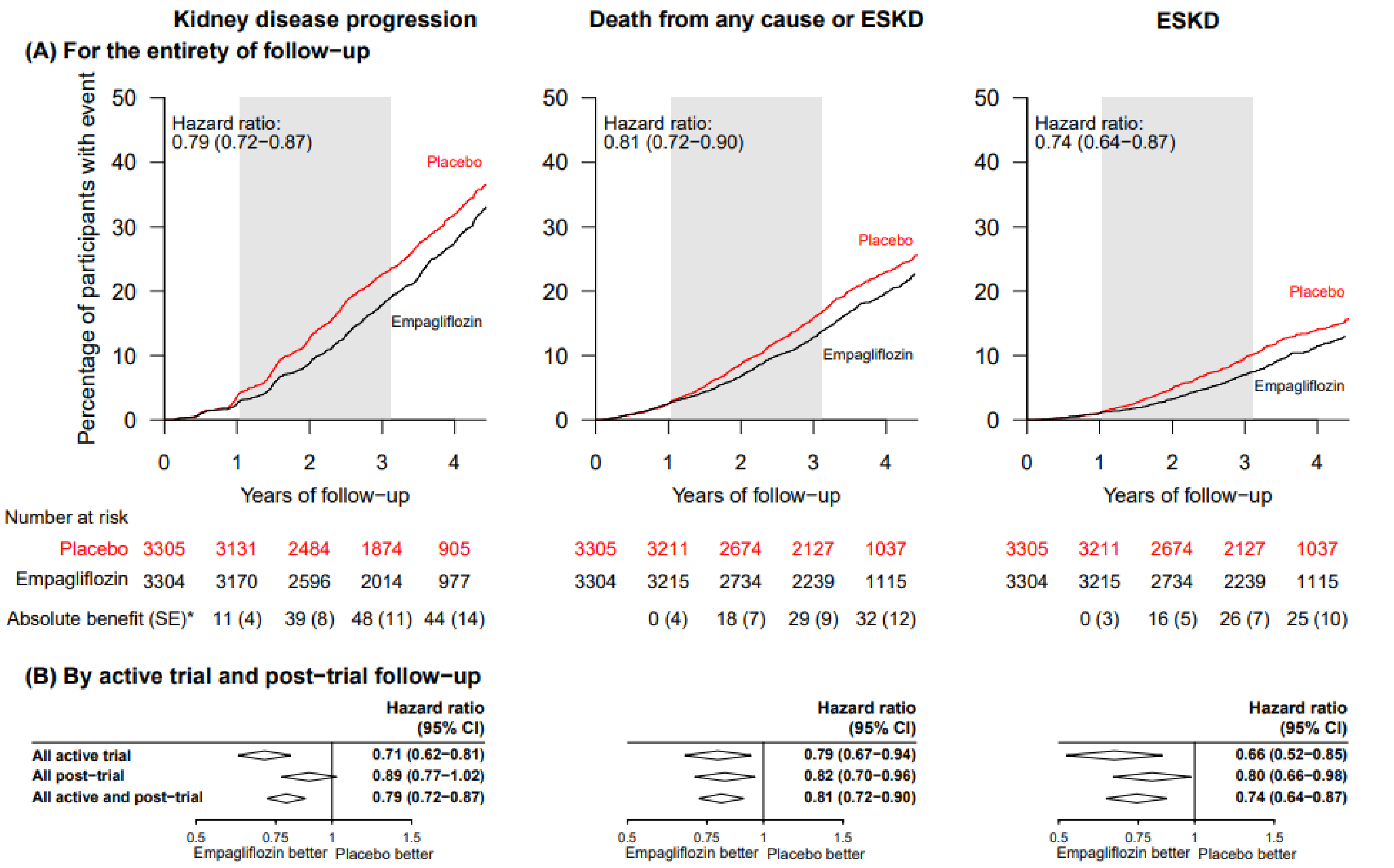


*Absolute difference in number of events per 1000 patients allocated to receive empagliflozin during the active trial period. A panels provide the KM-plots for the secondary outcomes for the entire follow-up period (active and post-trial periods combined). By contrast, B panels displays the hazard ratios in those originally allocated empagliflozin versus those originally allocated placebo separately for (a) the active trial, and (b) the post-trial period during which time no participant took study drug but some were started on non-trial SGLT2i (not necessarily empagliflozin). Presented analyses carry over the main results of the trial's primary outcome.

**Figure S4:** Effect of allocation to empagliflozin on progression of kidney disease over the entirety of follow-up by key pre−specified subgroups

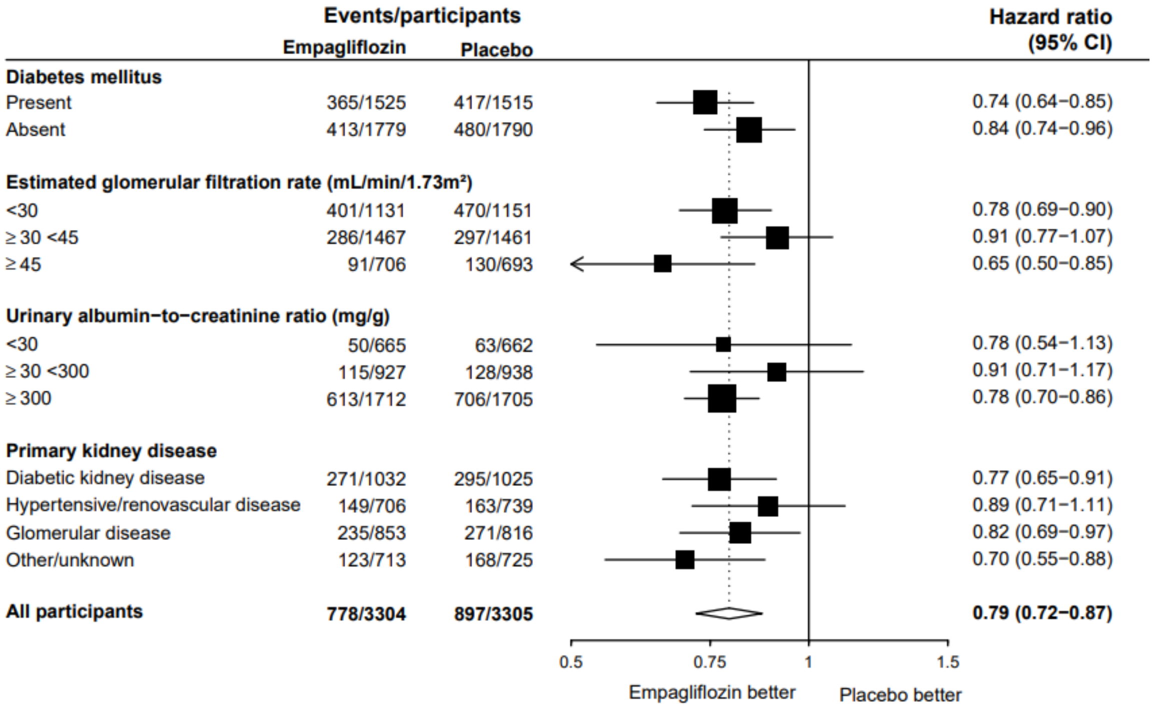


Entirety of follow-up is the active and post-trial periods combined. Presented analyses carry over the main results of the trial's primary outcome from its active period.

**Figure S5:** Effect of allocation to empagliflozin on cause−specific mortality over the entirety of follow-up

| Cause of death | Events: Empagliflozin (n=3304) | Events: Placebo (n=3305) | Hazard ratio (95% CI) |
|---|---|---|---|
| Coronary | 28 | 32 | 0.84 (0.51−1.40) |
| Other cardiac | 54 | 71 | 0.74 (0.52−1.05) |
| Stroke | 15 | 15 | 0.96 (0.47−1.96) |
| Other cardiovascular | 3 | 15 | 0.19 (0.06−0.66) |
| Presumed cardiovascular | 26 | 29 | 0.86 (0.51−1.47) |
| **Subtotal: Cardiovascular causes** | **126** | **162** | **0.75 (0.59−0.95)** |
| Renal disease | 13 | 10 | 1.24 (0.54−2.84) |
| Infection | 66 | 69 | 0.92 (0.66−1.29) |
| Cancer | 55 | 47 | 1.14 (0.77−1.68) |
| Other medical | 24 | 37 | 0.62 (0.37−1.04) |
| Non−medical causes | 17 | 11 | 1.49 (0.70−3.18) |
| **Subtotal: Non−cardiovascular causes** | **175** | **174** | **0.97 (0.79−1.20)** |
| **Total: Any cause** | **301** | **336** | **0.86 (0.74−1.01)** |

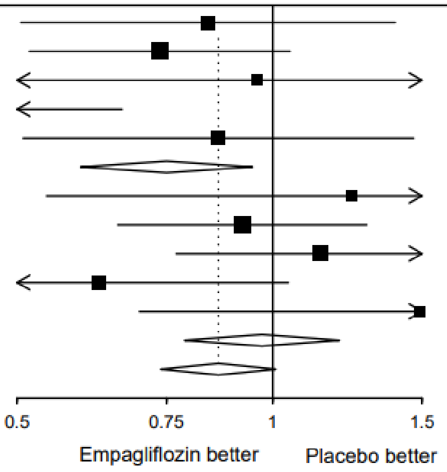

**Figure S6:** Effect of allocation to empagliflozin on estimated glomerular filtration rate over the entirety of follow−up

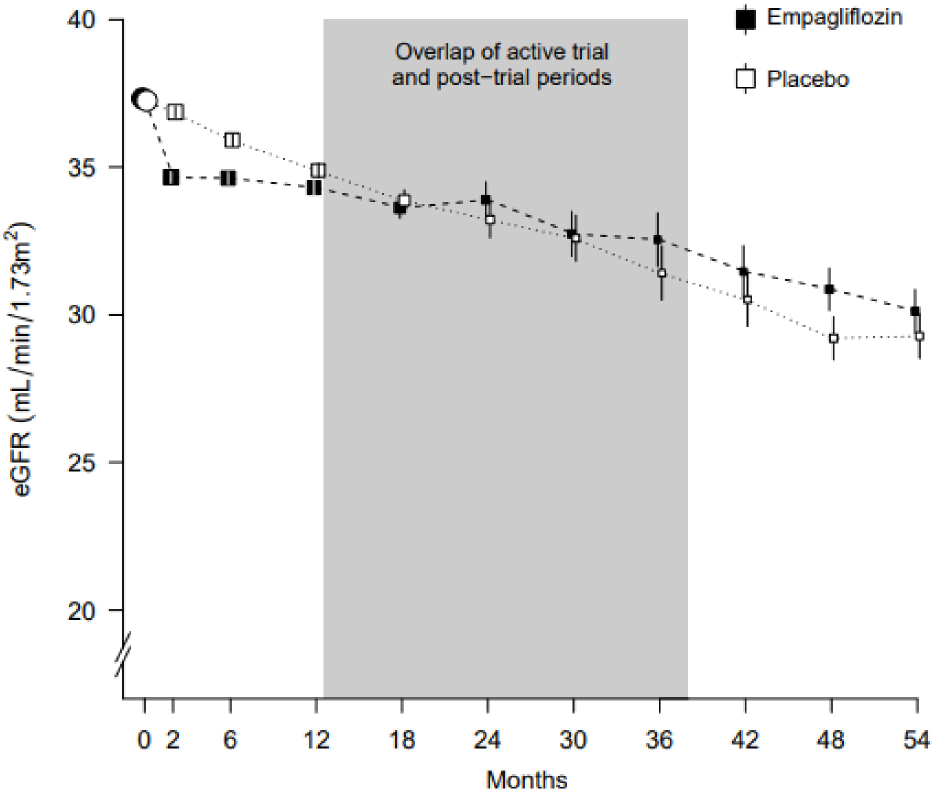


| Proportion taking an SGLT2 inhibitor (%)* | | | | | | | | | | |
|---|---|---|---|---|---|---|---|---|---|---|
| Empagliflozin | 95 | 95 | 93 | 92 | 89 | 81 | 60 | 43 | 50 | 55 |
| Placebo | 0 | 0 | 0 | 0 | 1 | 10 | 24 | 41 | 45 | 49 |

eGFR=estimated glomerular filtration rate. Plot of mean eGFR by time calculated from an MMRM model for the entirety of the trial with all analyses based on local creatinines (see supplementary statistical methods). The vertical lines indicate the 95% confidence intervals for the estimated means. The coordinates of the boxes are shifted slightly on the x axis to avoid overlap.

* Use of SGLT2 inhibitor based on reporting taking at least 80% of study drug or use of open-label SGLT2 inhibitor.

**Figure S7:** Effect of allocation to empagliflozin on absolute difference in mean estimated glomerular filtration rate at last local measurement by key pre−specified subgroups

| | Mean baseline eGFR (mL/min/1.73m²) | Number with last local measurement | Median (IQR) follow−up, years | Mean eGFR at last local measurement (mL/min/1.73m²) Empagliflozin | Placebo | Difference (95% CI) |
|---|---|---|---|---|---|---|
| **Diabetes** | | | | | | |
| Present | 35.4 | 3026 | 3.5 (2.3−4.4) | 30.3 (0.4) | 29.2 (0.4) | 1.2 (0.1,2.2) |
| Absent | 38.9 | 3564 | 3.6 (3.1−4.4) | 32.3 (0.3) | 31.7 (0.3) | 0.5 (−0.4,1.5) |
| **Estimated glomerular filtration rate (mL per minute per 1.73m²)** | | | | | | |
| <30 | 24.9 | 2274 | 3.6 (2.6−4.4) | 20.3 (0.4) | 19.6 (0.4) | 0.8 (−0.4,2.0) |
| ≥ 30 <45 | 36.7 | 2917 | 3.6 (2.8−4.4) | 31.1 (0.4) | 31.1 (0.4) | −0.0 (−1.1,1.0) |
| ≥ 45 | 58.7 | 1399 | 3.5 (2.3−4.2) | 49.6 (0.5) | 47.5 (0.5) | 2.1 (0.5,3.6) |
| **Urinary albumin−to−creatinine ratio (mg/g)** | | | | | | |
| <30 | 34.8 | 1321 | 3.6 (3.0−4.4) | 34.6 (0.5) | 33.6 (0.5) | 0.9 (−0.6,2.4) |
| ≥ 30 <300 | 36.2 | 1858 | 3.6 (2.5−4.4) | 32.7 (0.5) | 32.2 (0.5) | 0.5 (−0.7,1.8) |
| ≥ 300 | 38.9 | 3411 | 3.5 (2.4−4.3) | 29.4 (0.3) | 28.5 (0.3) | 0.9 (0.0,1.9) |
| **Primary kidney disease** | | | | | | |
| Diabetic kidney disease | 35.4 | 2049 | 3.5 (2.3−4.4) | 30.0 (0.4) | 28.8 (0.4) | 1.2 (0.0,2.5) |
| Hypertensive/renovascular disease | 35.1 | 1439 | 3.6 (2.9−4.4) | 30.5 (0.5) | 29.8 (0.5) | 0.7 (−0.7,2.2) |
| Glomerular disease | 42.9 | 1668 | 3.6 (3.2−4.3) | 33.9 (0.5) | 32.3 (0.5) | 1.6 (0.2,2.9) |
| Other/unknown | 35.7 | 1434 | 3.6 (2.5−4.4) | 31.2 (0.5) | 31.9 (0.5) | −0.7 (−2.2,0.8) |
| **All participants** | **37.3** | **6590** | **3.6 (2.6−4.4)** | **31.4 (0.2)** | **30.6 (0.2)** | **0.8 (0.1,1.4)** |

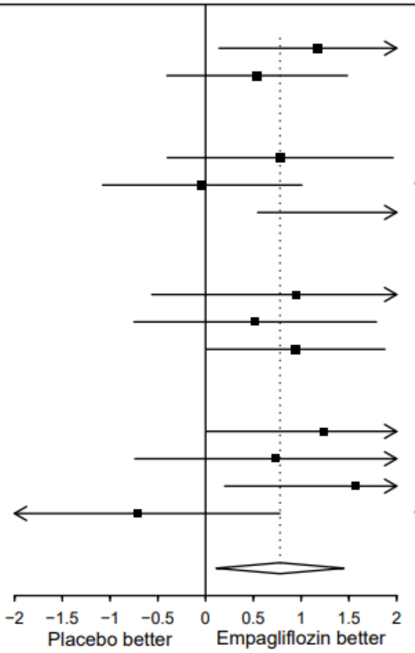


Last measurement defined as the last creatinine value recorded prior to death, end-stage kidney disease (i.e. date of commencement of maintenance dialysis or receipt of a kidney transplant), withdrawal of consent or end of follow-up. All analyses are based on local creatinine values.

## Supplementary Tables

**Table S1:** Representativeness of study participants

| **Category** | |
|---|---|
| Disease, problem, or condition under investigation | Chronic kidney disease (CKD) at risk of progression |
| Special considerations related to: | |
| Sex and gender | CKD affects men more than women, and the incidence (and prevalence) of kidney failure is greater in men than women. |
| Age | Prevalence of CKD increases steeply with age. |
| Race or ethnic group | The risks of developing CKD or its progression differ within countries by race and ethnicity. For example, Black, Hispanic and Native American people in the USA, Black and Asian people in the UK, and indigenous populations in Australia, Canada and South America are all at higher risk compared to the country's White population. |
| Geography | The crude prevalence of CKD varies globally from 2-15%, although this may in part be due to differences in methods of ascertainment. The causes of kidney disease vary substantially between countries. Diabetes causes between 10-55% of CKD; chronic glomerulonephritis causes between 5-40%. However, the cause of kidney disease is frequently unknown. |
| Other considerations | Previous large placebo-controlled trials of sodium glucose co-transporter-2 (SGLT2) inhibitors mainly recruited patients with type 2 diabetes and proteinuric CKD. Relatively few patients with CKD without diabetes were studied. The CREDENCE and SCORED trials exclusively studied patients with CKD with type 2 diabetes, and the DAPA-CKD trial in patients with proteinuric CKD studied 1398 patients without diabetes at baseline. Globally the majority of people with CKD have low levels of albuminuria (i.e. a urinary albumin-to-creatinine ratio less than 300 milligrams per gram) and do not have diabetes. |
| Overall representativeness of this trial | Participants in EMPA-KIDNEY were selected to have CKD at risk of progression, so do not represent the larger population of all people with CKD. Patients with polycystic kidney disease were excluded. Nevertheless, EMPA-KIDNEY included a wide range of patients with CKD, and the proportional effects of treatment are likely to be generalizable.<br><br>Biologic sex (male or female) was reported by the participants and used to calculate estimate glomerular filtration rate. Gender was not reported. The participants in the present trial demonstrated an expected higher number of men than women. In the post-trial cohort, the proportion of Black patients overall was 4%, but among patients enrolled in post-trial follow-up in North America, 17% were Black and 17% were of Hispanic ethnicity. These are similar to the total population distribution of the United States.<br><br>Causes of CKD, including diabetic kidney disease, were otherwise consistent with registry data where these were available from participating countries. EMPA-KIDNEY recruited patients from centres in Europe, North America and Asia. Post-trial follow-up did not include EMPA-KIDNEY participants from Japan. No patients were enrolled in Africa or Oceania. |

**Table S2:** Baseline characteristics at randomization for participants in post-trial follow-up (PTFU) and those not in PTFU

| | **Participants entering PTFU (n=4891)** | **Participants not known to have died at the end of active trial follow-up but did not enter PTFU (n=1362)** |
|---|---|---|
| **Demographics** | | |
| Age at randomization (years) | 63 (14) | 65 (13) |
| Sex | | |
| Men | 3227 (66%) | 929 (68%) |
| Women | 1664 (34%) | 433 (32%) |
| Race | | |
| White | 3055 (62%) | 554 (41%) |
| Black | 178 (4%) | 66 (5%) |
| Asian | 1582 (32%) | 725 (53%) |
| Mixed | 20 (0%) | 1 (0%) |
| Other | 56 (1%) | 16 (1%) |
| **Prior disease** | | |
| Prior diabetes* | 2107 (43%) | 683 (50%) |
| Prior cardiovascular disease§ | 1280 (26%) | 311 (23%) |
| **Clinical measurements** | | |
| Systolic blood pressure (mmHg) | 136.9 (18.3) | 134.8 (17.3) |
| Diastolic blood pressure (mmHg) | 78.6 (11.7) | 77.6 (11.8) |
| Body mass index (kg/m²) | 29.9 (6.6) | 28.8 (7.0) |
| **Laboratory measurements** | | |
| eGFR (mL/min/1.73m²)† | | |
| Mean (SD) | 36.9 (14.1) | 40.5 (16.0) |
| <30 | 1711 (35%) | 378 (28%) |
| ≥30 to <45 | 2210 (45%) | 575 (42%) |
| ≥45 | 970 (20%) | 409 (30%) |
| uACR (mg/g)† | | |
| Geometric mean (approx SE) | 213 (6) | 276 (14) |
| Median (Q1-Q3) | 317 (44-1063) | 393 (81-1085) |
| <30 | 1030 (21%) | 218 (16%) |
| ≥30 to ≤300 | 1363 (28%) | 380 (28%) |
| >300 | 2498 (51%) | 764 (56%) |
| **Concomitant medication use** | | |
| RAS inhibitor | 4208 (86%) | 1141 (84%) |
| Any diuretic | 2080 (43%) | 509 (37%) |
| Any lipid-lowering medication | 3220 (66%) | 878 (64%) |
| **Cause of kidney disease** | | |
| Diabetic kidney disease | 1404 (29%) | 472 (35%) |
| Hypertensive/renovascular disease | 1125 (23%) | 247 (18%) |
| Glomerular disease | 1306 (27%) | 342 (25%) |
| Other/unknown | 1056 (22%) | 301 (22%) |
| **5 year predicted kidney failure risk (%)** | | |
| Median (Q1-Q3) | 10 (3-29) | 7 (2-25) |

Participants who died during the active trial period (n=317) or withdrew consent during the active trial period (n=39) not shown. Figures are n (%), mean (SD), geometric mean (approx SE) or median (Q1-Q3). eGFR=estimated glomerular filtration rate. uACR=urinary albumin-to-creatinine ratio. RAS=renin-angiotensin system. * Prior diabetes mellitus defined as participant-reported history of diabetes of any type, use of glucose-lowering medication or baseline HbA1c ≥48 mmol/mol at Randomization visit. § Defined as self-reported history of myocardial infarction, heart failure, stroke, transient ischemic attack, or peripheral arterial disease. † Uses central measurement taken at the randomization visit, or more recent local laboratory result before randomization. Those with missing data for BMI (n=15) not presented in relevant rows.

**Table S3:** Baseline characteristics at the last follow-up during the active trial treatment phase for participants entering post-trial follow-up (PTFU) and those not in PTFU and by use of SGLT2 inhibitors (SGLT2i) at the end of PTFU

| | **Characteristics of participants as they entered PTFU** | | | **Participants not known to have died at the end of active trial follow-up but did not enter PTFU (n=1362)** |
|---|---|---|---|---|
| | **Participants recorded as taking SGLT2i at last PTFU visit (n=2050)** | **Participants not recorded as taking SGLT2i at last PTFU visit (n=2841)** | **All participants (n=4891)** | |
| **Demographics** | | | | |
| Age (years) | 65 (14) | 64 (14) | 65 (14) | 67 (13) |
| Sex | | | | |
| Men | 1388 (68%) | 1839 (65%) | 3227 (66%) | 929 (68%) |
| Women | 662 (32%) | 1002 (35%) | 1664 (34%) | 433 (32%) |
| Region | | | | |
| Europe | 1239 (60%) | 1143 (40%) | 2382 (49%) | 111 (8%) |
| North America | 424 (21%) | 632 (22%) | 1056 (22%) | 539 (40%) |
| China or Malaysia | 387 (19%) | 1066 (38%) | 1453 (30%) | 124 (9%) |
| Japan | 0 (0%) | 0 (0%) | 0 (0%) | 588 (43%) |
| Race | | | | |
| White | 1491 (73%) | 1564 (55%) | 3055 (62%) | 554 (41%) |
| Black | 63 (3%) | 115 (4%) | 178 (4%) | 66 (5%) |
| Asian | 461 (22%) | 1121 (39%) | 1582 (32%) | 725 (53%) |
| Mixed | 11 (<1%) | 9 (<1%) | 20 (<1%) | 1 (<1%) |
| Other | 24 (1%) | 32 (1%) | 56 (1%) | 16 (1%) |
| **Prior disease** | | | | |
| Prior diabetes* | 959 (47%) | 1236 (44%) | 2195 (45%) | 706 (52%) |
| Prior cardiovascular disease§ | 600 (29%) | 813 (29%) | 1413 (29%) | 342 (25%) |
| **Clinical measurements** | | | | |
| Systolic blood pressure (mmHg) | | | | |
| Mean (SD) | 131.8 (18.0) | 134.7 (19.0) | 133.4 (18.6) | 131.3 (17.3) |
| Missing | 68 (3%) | 269 (9%) | 337 (7%) | 158 (12%) |
| Diastolic blood pressure (mmHg) | | | | |
| Mean (SD) | 76.9 (11.4) | 77.0 (12.3) | 77.0 (11.9) | 75.0 (11.1) |
| Missing | 68 (3%) | 269 (9%) | 337 (7%) | 158 (12%) |
| Body mass index (kg/m²) | | | | |
| Mean (SD) | 30.0 (6.5) | 28.8 (6.4) | 29.3 (6.5) | 28.0 (7.0) |
| Missing | 73 (4%) | 272 (10%) | 345 (7%) | 160 (12%) |
| **Laboratory measurements** | | | | |
| eGFR (mL/min/1.73m²)† | | | | |
| Mean (SD) | 35.2 (13.7) | 30.9 (15.2) | 32.8 (14.7) | 36.0 (16.3) |
| On kidney failure replacement therapy | 8 (<1%) | 174 (6%) | 182 (4%) | 43 (3%) |
| <20 | 124 (6%) | 580 (20%) | 704 (14%) | 170 (12%) |
| ≥20 to <30 | 681 (33%) | 761 (27%) | 1442 (29%) | 308 (23%) |
| ≥30 to <45 | 858 (42%) | 758 (27%) | 1616 (33%) | 408 (30%) |
| ≥45 | 318 (16%) | 341 (12%) | 659 (13%) | 285 (21%) |
| Missing | 61 (3%) | 227 (8%) | 288 (6%) | 148 (11%) |
| uACR (mg/g)† | | | | |
| Geometric mean (approx SE) | 249 (10) | 313 (12) | 283 (8) | 356 (17) |
| Median (Q1-Q3) | 320 (78-902) | 391 (70-1232) | 351 (73-1066) | 445 (118-1204) |
| <30 | 237 (12%) | 287 (10%) | 524 (11%) | 92 (7%) |
| ≥30 to ≤300 | 639 (31%) | 731 (26%) | 1370 (28%) | 364 (27%) |
| >300 | 911 (44%) | 1201 (42%) | 2112 (43%) | 644 (47%) |
| Missing | 255 (12%) | 448 (16%) | 703 (14%) | 219 (16%) |
| **Concomitant medication use** | | | | |
| RAS inhibitor | 1775 (87%) | 2137 (75%) | 3912 (80%) | 1087 (80%) |
| Any diuretic | 497 (24%) | 512 (18%) | 1009 (21%) | 264 (19%) |
| Any lipid-lowering medication | 1460 (71%) | 1893 (67%) | 3353 (69%) | 908 (67%) |

| | Characteristics of participants as they entered PTFU | | | Participants not known to have died at the end of active trial follow-up but did not enter PTFU (n=1362) |
|---|---|---|---|---|
| | **Participants recorded as taking SGLT2i at last PTFU visit (n=2050)** | **Participants not recorded as taking SGLT2i at last PTFU visit (n=2841)** | **All participants (n=4891)** | |
| **Cause of kidney disease** | | | | |
| Diabetic kidney disease | 583 (28%) | 821 (29%) | 1404 (29%) | 472 (35%) |
| Hypertensive/renovascular disease | 417 (20%) | 708 (25%) | 1125 (23%) | 247 (18%) |
| Glomerular disease | 604 (29%) | 702 (25%) | 1306 (27%) | 342 (25%) |
| Other/unknown | 446 (22%) | 610 (21%) | 1056 (22%) | 301 (22%) |
| **5 year predicted risk of kidney failure (%)‡** | | | | |
| Median (Q1-Q3) – overall | 12 (5-30) | 22 (5-63) | 15 (5-45) | 13 (3-41) |
| Median (Q1-Q3) – empagliflozin group | 12 (5-28) | 20 (5-61) | 15 (5-42) | 12 (3-28) |
| Median (Q1-Q3) – placebo group | 12 (5-32) | 23 (6-64) | 16 (5-47) | 14 (3-46) |

Participants who died during the active trial period (n=317) or withdrew consent during the active trial period (n=39) not shown. Figures are n (%), mean (SD), geometric mean (approx SE) or median (Q1-Q3). eGFR=estimated glomerular filtration rate. uACR=urinary albumin-to-creatinine ratio. RAS=renin-angiotensin system.

* Prior diabetes mellitus defined as diabetes at randomization or incident diabetes reported during the trial. § Defined as self-reported history of myocardial infarction, heart failure, stroke, transient ischemic attack, or peripheral arterial disease, or incident myocardial infarction, heart failure, stroke, transient ischemic attack, or peripheral arterial disease reported during the active trial. † Uses central measurement taken at the final follow-up visit among those not on kidney replacement therapy at final follow-up visit. ‡ Not calculated for those already on kidney replacement therapy at final follow-up visit.

Median (Q1-Q3) 5-year predicted kidney failure risk (%) at randomization among those participants recorded as taking SGLT2i at last PTFU visit in empagilflozin group 7 (3-19) and placebo group 7 (2-17). Median (Q1-Q3) 5-year predicted kidney failure risk (%) at randomization among those participants who were not on an SGLT2i at last PTFU visit in empagilflozin group 13 (4-39) and placebo group 14 (4-42).

3 vs 4 participants allocated empagliflozin and placebo respectively were unblinded during the active trial period, and 15 vs 16 participants allocated empagliflozin and placebo respectively were unblinded prior to the end of PTFU.

**Table S4:** Use of RAS inhibitors and mineralocorticoid receptor antagonists over time

| | **Renin-angiotensin system inhibitor use** | | **Mineralocorticoid receptor antagonist use** | |
|---|---|---|---|---|
| | **Empagliflozin** | **Placebo** | **Empagliflozin** | **Placebo** |
| **All participants** | N=3304 | N=3305 | N=3304 | N=3305 |
| Active trial period | | | | |
| 12 months | 2610/3164 (82%) | 2608/3159 (83%) | 195/3164 (6%) | 233/3159 (7%) |
| 24 months | 1541/1884 (82%) | 1545/1875 (82%) | 119/1884 (6%) | 147/1875 (8%) |
| 36 months | 273/326 (84%) | 248/323 (77%) | 19/326 (6%) | 25/323 (8%) |
| Study average | 82% | 82% | 6% | 8% |
| **Participants entering post-trial follow-up** | N=2472 | N=2419 | N=2472 | N=2419 |
| Active trial period | | | | |
| 12 months | 2025/2423 (84%) | 1954/2363 (83%) | 148/2423 (6%) | 180/2363 (8%) |
| 24 months | 1220/1483 (82%) | 1161/1417 (82%) | 101/1483 (7%) | 113/1417 (8%) |
| 36 months | 249/297 (84%) | 225/289 (78%) | 17/297 (6%) | 24/289 (8%) |
| Study average | 83% | 82% | 6% | 8% |
| Post-trial period | | | | |
| 12 months | 1525/2186 (70%) | 1484/2147 (69%) | 125/2186 (6%) | 138/2147 (6%) |
| 24 months | 1582/2376 (67%) | 1524/2312 (66%) | 156/2376 (7%) | 157/2312 (7%) |
| Study average | 68% | 68% | 6% | 7% |

Active trial periods defined using 12, 24, and 36 months follow-up visit windows. Active trial period denominators are those known to be alive in each period. Post-trial periods defined using information nearest to 12 and 24 months since completion of active trial follow-up. Post-trial period denominators are those who joined post-trial follow-up, had a follow-up in the period and were known to be alive in the relevant period. Study average use calculated using weights proportional to the total person years at risk in each year.

**Table S5:** Effect of allocation to empagliflozin on components of primary outcome over the entirety of follow-up (i.e. combining active trial and post-trial follow-up periods)

| | Empagliflozin (N=3304) | | Placebo (N=3305) | | |
|---|---|---|---|---|---|
| | **Participants with event** | **No. of events per 100 patient years** | **Participants with event** | **No. of events per 100 patient years** | **Hazard ratio (95% CI)** |
| **Primary outcome: progression of kidney disease or death from CV causes** | 865 (26.2%) | 8.4 | 1001 (30.3%) | 10.0 | 0.79 (0.72-0.87) |
| End stage kidney disease (commencement of maintenance dialysis or receipt of kidney transplant) | 296 (9.0%) | 2.7 | 372 (11.3%) | 3.5 | 0.74 (0.64-0.87) |
| Sustained decline in eGFR to <10ml/min/1.73m² | 247 (7.5%) | 2.2 | 300 (9.1%) | 2.8 | 0.77 (0.65-0.91) |
| Renal death (i.e. death from kidney failure) | 13 (0.4%) | 0.1 | 10 (0.3%) | 0.1 | 1.24 (0.54-2.84) |
| Sustained decline of ≥40% in eGFR from randomization | 726 (22.0%) | 7.0 | 828 (25.1%) | 8.2 | 0.80 (0.72-0.88) |
| Death from cardiovascular cause | 126 (3.8%) | 1.1 | 162 (4.9%) | 1.5 | 0.75 (0.59-0.95) |

Presented analyses carry over the main results of the trial's primary outcome from the active trial period. A sensitivity analysis of the primary outcome analyses using only local estimated GFR measurements collected throughout the entire follow−up period which did not differentiate the active trial versus post−trial periods (i.e. the last locally−measured estimate GFR recorded during PTFU was considered to be a “sustained” decline) found: 842 (25.5%) vs 976 (29.5%); HR=0.79 (95% CI 0.72−0.86).

**Table S6:** Absolute benefits of allocation to empagliflozin on primary and secondary outcomes over the entirety of follow-up

| | Absolute difference in number of events per 1000 patients allocated to empagliflozin | | | |
|---|---|---|---|---|
| | **Kidney disease progression or CV death** | **Kidney disease progression** | **Death from any cause or ESKD** | **ESKD** |
| **Active trial period only** | | | | |
| End of year 1 | 12 (5) | 11 (4) | 0 (4) | 0 (3) |
| End of year 2 | 43 (10) | 42 (9) | 19 (8) | 17 (6) |
| End of active trial period (2.5 years) | 57 (14) | 56 (14) | 25 (11) | 26 (8) |
| **Post-trial period only** | | | | |
| End of year 1 | 15 (8) | 14 (7) | 10 (7) | 8 (6) |
| End of year 2 | 25 (13) | 19 (13) | 19 (10) | 12 (9) |
| **Active trial and post-trial periods combined** | | | | |
| End of year 1 | 12 (5) | 11 (4) | 0 (4) | 0 (3) |
| End of year 2 | 41 (8) | 39 (8) | 18 (7) | 16 (5) |
| End of year 3 | 52 (11) | 48 (11) | 29 (9) | 26 (7) |
| End of year 4 | 45 (14) | 44 (14) | 32 (12) | 25 (10) |

CV=cardiovascular. ESKD=end-stage kidney disease (i.e. date of commencement of maintenance dialysis or receipt of a kidney transplant).
